# Targeting engulfment and cell motility 1 protein methylation attenuates M2 macrophage infiltration and boosts anti-PD-1 efficacy in colorectal cancer

**DOI:** 10.1093/gastro/goag056

**Published:** 2026-06-19

**Authors:** Guanman Li, Zexian Chen, Kai Wang, Junguo Chen, Xiao Yue, Tianze Huang, Bin Zhang, Danling Liu, Shuang Guo, Jiong Bi, Ping Lan, Xiaosheng He

**Affiliations:** Guangdong Institute of Gastroenterology, Guangdong Provincial Key Laboratory of Colorectal and Pelvic Floor Diseases, Department of Colorectal Surgery, The Sixth Affiliated Hospital, Sun Yat-sen University, Guangzhou, Guangdong 510510, P. R. China; School of Medicine (Shenzhen), Sun Yat-sen University, Shenzhen, Guangdong 518107, P. R. China; Guangdong Institute of Gastroenterology, Guangdong Provincial Key Laboratory of Colorectal and Pelvic Floor Diseases, Department of Colorectal Surgery, The Sixth Affiliated Hospital, Sun Yat-sen University, Guangzhou, Guangdong 510510, P. R. China; Department of Anaesthesia, The Sixth Affiliated Hospital, Sun Yat-Sen University, Guangzhou, Guangdong 510510, P. R. China; Guangdong Institute of Gastroenterology, Guangdong Provincial Key Laboratory of Colorectal and Pelvic Floor Diseases, Department of Colorectal Surgery, The Sixth Affiliated Hospital, Sun Yat-sen University, Guangzhou, Guangdong 510510, P. R. China; Department of Thoracic Surgery, Thoracic Cancer Center, The Sixth Affiliated Hospital, Sun Yat-sen University, Guangzhou, Guangdong 510510, P. R. China; Guangdong Institute of Gastroenterology, Guangdong Provincial Key Laboratory of Colorectal and Pelvic Floor Diseases, Department of Colorectal Surgery, The Sixth Affiliated Hospital, Sun Yat-sen University, Guangzhou, Guangdong 510510, P. R. China; Department of Colorectal Surgery, The Second Affiliated Hospital of South China University of Technology, Guangzhou, Guangdong 510510, P. R. China; Guangdong Institute of Gastroenterology, Guangdong Provincial Key Laboratory of Colorectal and Pelvic Floor Diseases, Department of Colorectal Surgery, The Sixth Affiliated Hospital, Sun Yat-sen University, Guangzhou, Guangdong 510510, P. R. China; Guangdong Institute of Gastroenterology, Guangdong Provincial Key Laboratory of Colorectal and Pelvic Floor Diseases, Department of Colorectal Surgery, The Sixth Affiliated Hospital, Sun Yat-sen University, Guangzhou, Guangdong 510510, P. R. China; Guangdong Institute of Gastroenterology, Guangdong Provincial Key Laboratory of Colorectal and Pelvic Floor Diseases, Department of Colorectal Surgery, The Sixth Affiliated Hospital, Sun Yat-sen University, Guangzhou, Guangdong 510510, P. R. China; Guangdong Institute of Gastroenterology, Guangdong Provincial Key Laboratory of Colorectal and Pelvic Floor Diseases, Department of Colorectal Surgery, The Sixth Affiliated Hospital, Sun Yat-sen University, Guangzhou, Guangdong 510510, P. R. China; Laboratory of General Surgery, The First Affiliated Hospital, Sun Yat-Sen University, Guangzhou, Guangdong 510080, P. R. China; Guangdong Institute of Gastroenterology, Guangdong Provincial Key Laboratory of Colorectal and Pelvic Floor Diseases, Department of Colorectal Surgery, The Sixth Affiliated Hospital, Sun Yat-sen University, Guangzhou, Guangdong 510510, P. R. China; Guangdong Institute of Gastroenterology, Guangdong Provincial Key Laboratory of Colorectal and Pelvic Floor Diseases, Department of Colorectal Surgery, The Sixth Affiliated Hospital, Sun Yat-sen University, Guangzhou, Guangdong 510510, P. R. China

**Keywords:** ELMO1, immunotherapy, M2 macrophages, protein methylation, CCL18

## Abstract

**Background:**

Immunotherapy has been very successful in the field of cancer treatment. However, some patients exhibit unresponsive to PD-1 blockade, even for microsatellite instability-high (MSI-H) colorectal cancer (CRC).

**Methods:**

Engulfment and cell motility 1 (ELMO1) expression and macrophage infiltration in clinical samples and mouse models were detected by using quantitative real-time polymerase chain reaction (qRT-PCR) and immunohistochemistry staining. Western blotting, qRT-PCR, and enzyme-linked immunosorbent assay (ELISA) were used to evaluate ELMO1, C-C chemokine ligand 18 (CCL18), and transforming growth factor-β (TGF-β) expression in tumor cells. Macrophage phenotypes were determined using qRT-PCR, flow cytometry, and RNA sequencing. Tumor viability was investigated using XTT assays and foci formation assays.

**Results:**

ELMO1 was significantly overexpressed in MSI-H CRC and associated with poor clinical outcomes. *ELMO1* depletion suppressed the growth of MSI-H cancer cells but exerted minimal effects on microsatellite stable (MSS) CRC cells. Tumor cell-shed ELMO1 recruited M2 macrophages and facilitated M0-to-M2 polarization by signal transducer and activator of transcription 6 (STAT6) activation. Accumulated M2 macrophages further promoted the proliferation of ELMO1-positive tumor cells. Mechanistically, CCL18 secreted by M2 macrophages strengthened the binding between protein arginine methyltransferase 5 (PRMT5) and ELMO1. Pharmacological inhibition of PRMT5 reduced ELMO1 methylation, thereby decreasing the level of extracellular ELMO1. Moreover, PRMT5 blockade diminished M2 macrophage infiltration, inhibited tumor growth, and improved the responsiveness of ELMO1-overexpressing MSI-H CRC to anti-PD-1 immunotherapy.

**Conclusions:**

Our findings demonstrate that ELMO1 acts as a novel biomarker to classify the MSI-H group into distinct subtypes and provides a promising therapeutic target for the treatment of ELMO1-overexpressing MSI-H CRC.

## Introduction

Colorectal cancer (CRC) is the third most common cancer worldwide, with more than 1.8 million new cases reported globally and approximately 560,000 new cases annually in China [1]. Immune checkpoint inhibitors (ICIs) have shown beneficial effects to patients with microsatellite instability-high (MSI-H) [2, 3]. Nevertheless, more than half of MSI-H CRC patients exhibit primary or secondary resistance to ICIs, and some develop tumor progression in the course of disease [4–6]. The reasons behind this lack of response in some patients remain unclear.

The tumor environment contains various immune cells, which interact in intricate ways that can either promote or hinder tumor growth [7]. Tumor-associated macrophages (TAMs), especially the M2 subtype, are important components of the tumor microenvironment, which can release a variety of anti-inflammatory cytokines and chemokines, such as C-C chemokine ligand 18 (CCL18), to promote tumor growth, angiogenesis, metastasis, and tumor immune escape [8]. M2-like TAMs can express T cell immune checkpoint ligands (such as programmed death-ligand 1 [PD-L1] and programmed death-ligand 2 [PD-L2]), secrete inhibitory cytokines (such as interleukin [IL]-10 and transforming growth factor-β [TGF-β]), and recruit immunosuppressive cells (such as regulatory T [Treg] cells) to suppress anti-tumor immune response [9–11]. Accumulating evidence has demonstrated that the immune escape of CRC is predominantly modulated by the composition and distribution of immune cells within the tumor microenvironment, as well as regulatory molecular factors in tumor cells [12]. Hence, it is crucial to identify key molecules that mediate the crosstalk between tumor cells and immune cells.

It has been reported that engulfment and cell motility protein 1 (ELMO1) plays a critical role in macrophage-associated pathological processes, including bacterial infection, inflammatory disorders, and tumor progression [13–16]. By interacting with dedicator of cytokinesis (DOCK) family proteins, ELMO1 modulates cell migration and invasion to improve tumor progression [17]. In upper gastrointestinal tumors and certain benign diseases, ELMO1 serves as a key mediator regulating the crosstalk between tumor cells and immune cells [18–22]. However, the correlation between ELMO1 and CRC progression, as well as the relevant regulatory mechanism, still remains unclear.

In this study, we discovered a novel association between ELMO1 and MSI-H tumor microenvironment remodeling. We found that high ELMO1 expression was associated with a worse prognosis in MSI-H CRC group. Silencing *ELMO1* suppressed cell growth in MSI-H cancer cells. ELMO1 shedding from tumor cells induced M2 macrophages accumulation and facilitated M0-to-M2 polarization by activating signal transducer and activator of transcription 6 (STAT6) signaling. Moreover, CCL18 secreted by M2 macrophages enhanced the interaction between protein arginine methyltransferase 5 (PRMT5) and ELMO1. Pharmacological inhibition of PRMT5 reduced ELMO1 methylation and suppressed tumor cell growth. Additionally, the combination of PRMT5 inhibitor and anti-PD1 antibody enhanced the antitumor effect on tumors with high ELMO1 expression, which clarified the crosstalk between CRC and TAMs. Collectively, our results provide a promising therapeutic target for the treatment of ELMO1-overexpressing MSI-H CRC.

## Materials and methods

### Clinical samples

CRC patients who underwent radical resection from 2014 to 2019 at the Sixth Affiliated Hospital, Sun Yat-Sen University (Guangdong, China), were enrolled in this study. Pathological diagnosis and staging of CRC were performed by the WHO Classification of Tumors of the Digestive System 4th Edition and American Joint Committee on Cancer (AJCC) Staging System. A total of 80 fresh CRC tissue specimens were collected and subjected to quantitative real-time polymerase chain reaction (qRT-PCR) detection for gene expression analysis (summarized in Supplementary Table S1). Another independent cohort of 72 paraffin-embedded CRC tissues was enrolled for immunohistochemical staining and subsequent clinical correlation analysis, with detailed clinical characteristics listed in Table 1 and Supplementary Table S2. Samples used in this study were approved by the Committees for Ethical Review of Research involving human subjects at the Sixth Affiliated Hospital, Sun Yat-Sen University (No. 2022ZSLYEC-172 and No. 2023ZSLYEC-135).

**Table 1:** The association between ELMO1 expression and clinicopathological features in 72 CRCs

Clinicopathological features	Total case	High expression of ELMO1	*P* value
No. of patients	72	18 (25%)	
Age (mean 46 years)			0.801
≤46	41	12 (29.3%)	
>46	31	6 (19.4%)	
Sex			0.35
Male	40	9 (22.5%)	
Female	32	9 (28.2%)	
MSI status			**0.017**
MSI-H	35	16 (45.7%)	
MSS	37	2 (5.4%)	
Tumor stage			0.237
pT1–pT2	13	1 (7.7%)	
pT3–pT4	59	17 (28.8%)	
Lymph-node metastasis			**0.031**
pN0	54	12 (22.2%)	
pN+	18	6 (33.3%)	
Organ metastasis			0.256
M0	61	12 (19.7%)	
M1	11	6 (54.5%)	
TNM stage			**0.025**
I–II	51	9 (17.6%)	
III–IV	21	9 (42.9%)	

Bold values indicate statistically significant differences (*P* < 0.05).

Five markers (BAT-26, BAT-25, D5S346, D2S123, and D17S250) that are recommended by National Cancer Institute for the uniform analysis of MSI-H in CRC were evaluated by qRT-PCR of genomic DNA isolated from patients.

### Cell culture

Colorectal cell lines HCT116 (CCL-247), DLD-1 (CCL-221), SW480 (CCL-228), HT-29 (HTB-38), Caco2 (HTB-37), RKO (CRL-2577), LoVo (CCL-229), and normal colonic epithelial cell lines HIEC6 (CRL-3266) were purchased from the American Type Culture Collection (ATCC; Rockefeller, MD, USA). Cells were maintained in DMEM-high glucose (C11995500BT; Gibco; Waltham, MA, USA) supplemented with 10% fetal bovine serum (FBS; FSP500; ExCell Bio; Suzhou, Jiangsu, China) and 1% penicillin-streptomycin (15140–122; Gibco) in a humidified atmosphere of 5% CO_2_ at 37°C. THP-1 cells were obtained from China Center for Type Culture Collection and cultured in RPMI1640 (12633020; Gibco) with 10% heat-inactivated FBS, 1% penicillin-streptomycin, and 0.05 mM β-mercaptoethanol (60–24-2; TCI; Shanghai, China).

### Macrophages polarization

THP-1 cells were differentiated into an M0 stage with the stimulus of 1 µg/mL phorbol 12-myristate 13-acetate (PMA; 524400; Merck Sigma; MA, USA). Then, the M0 macrophages were polarized into M2 macrophages via IL-4 (20 ng/mL; 200–04-20ug; Thermo Fisher; Waltham, MA, USA) and IL-13 (20 ng/mL; 200–13-10ug; Thermo Fisher) stimulation.

### Macrophage migration assay

After *in vitro* polarization of THP-1 cells, the migration assay was performed using 24-well plates with 5.0 μm pore inserts. The indicated tumor cells were placed on the bottom of the lower chamber as a chemoattractant and M0 or M2 macrophages were added to the upper transwell inserts (14331; Labselect; Beijing, China) and incubated for 48 h at 37°C and 5% CO_2_. To inhibit protein secretion, tumor cells were incubated with 10 μM monensin for 48 h prior to the migration assay. For the M2 macrophage migration assay induced with the ELMO1 recombinant (P05836; Solarbio; Beijing, China), M2 macrophages were plated in the upper inserts, and ELMO1 recombinant was added to the bottom wells. After 48 h, the transwell inserts were removed from the plate and washed three times with PBS. Then, the migrated cells attached to the lower surface of the membrane were fixed with 4% paraformaldehyde solution (P0099; Beyotime Biotechnology; Shanghai, China), stained with 0.1% crystal violet (C0121; Beyotime Biotechnology) and then quantified by counting the cell number at ten random fields under a microscope.

### Transwell co-culture assay of M0 macrophages and tumor cells

Indirect co-culture assay was performed using 3.0 μm cell culture inserts (14122; Labselect). M0-polarized THP-1 cells were seeded in the upper insert, and indicated tumor cells were seeded into the bottom wells in the presence of PMA. Macrophages were then collected and analyzed M2 macrophage markers (CD68 [333806; BioLegend; San Diego, CA, USA] and CD163 [326511; BioLegend]) by flow cytometry and qPCR.

### Co-immunoprecipitation

Co-immunoprecipitation (Co‑IP) assay was performed to detect the protein interaction between PRMT5 and ELMO1. Briefly, cells expressing Flag-ELMO1 were lysed in 50 mM Tris-HCl (pH 7.5), 150 mM NaCl, 5 mM EDTA (pH 8.0), 1% NP-40, 0.5% deoxycholate, 0.1% SDS, and Roche complete protease inhibitor mixture (Roche, UK) and incubated with indicated antibodies (anti-Flag; TT0003; Abmart; Shanghai, China) at 4°C for 1 h, and then incubated with Protein A/G magnetic beads. After extensive washing, bound proteins were eluted and analyzed by Western blotting. Input samples were simultaneously loaded as internal controls for immunoblotting detection.

### Animal models

All animal experiments were approved by and performed in accordance with the Committee of the Use of Live Animals in Teaching and Research at Sun Yat-sen University. For *in vivo* tumorigenicity assay, 4-week-old male nude mice were engrafted with CRC cells in the back of the right hindlimb by subcutaneously injecting different numbers of cells (HCT116: 5 × 10^5^; DLD-1: 2 × 10^6^) in a 100 μL suspension. After 2 weeks, 20 μL concentrated M2 conditioned medium (CM) and serum-free medium (control group) were injected into the tumor mass subcutaneously separately every 3 days. Tumor volume was measured every 3 days over a 4-week period. The tumor volume was measured weekly and calculated using the formula V = 0.5 × W^2^ × L (V, volume; L, length; W, width). All animal studies were conducted in compliance with animal protocols approved by the Animal Care and Ethics Committee of Sun Yat-sen University and followed the National Health Guidelines on the Care and Use of Animals (IACUC-2020070602, IACUC-2022031401, IACUC-2022031402).

### Statistical analysis

Transcriptomic analysis was performed for exploratory candidate screening, and key genes were further validated by independent functional experiments to exclude false positives. For clinical data, demographic characteristics were shown using descriptive statistics. The correlation between ELMO1 expression and clinical pathological features was assessed using Pearson’s chi-square test. Survival curves were derived using the Kaplan-Meier method and assessed univariately using the log-rank test, with a significance level set at two-sided 0.05. The above steps were performed using the statistical software SPSS version 20.0. The analysis of 22 types of immune cell infiltration was conducted using the CIBERSORT algorithm [23]. Data from cell proliferation, colony formation, Western blot, and qPCR assays were analyzed using Student’s *t*-test and one‑way analysis of variance (ANOVA). The student’s *t*-test was used for two-group comparisons, while one-way ANOVA followed by Tukey’s post hoc test was applied for multiple-group comparisons. All statistical analyses were performed using SPSS and GraphPad Prism. A *P* value < 0.05 was considered statistically significant.

## Results

### ELMO1 is overexpressed in MSI-H CRC and associated with poor prognosis

**Figure 1:**
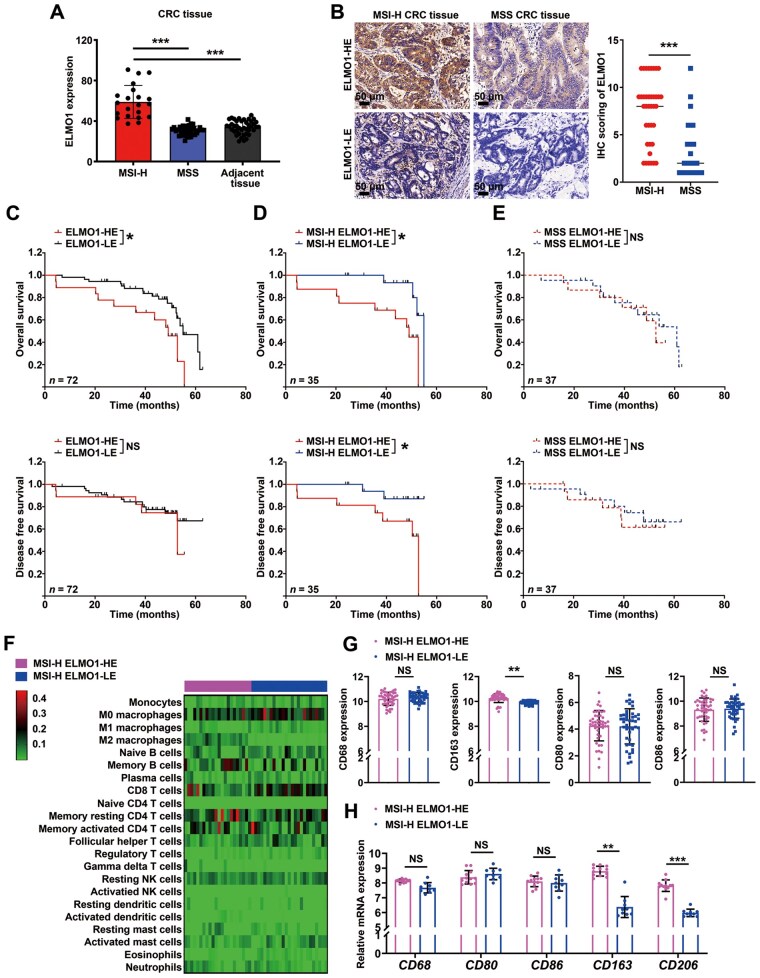
Overexpression of ELMO1 correlated with poor survival outcomes in MSI-H CRC patients. (A) The relative expression of ELMO1 in 80 CRC tumor and 41 non-tumor samples was detected using qPCR (***P < 0.0001). (B) Expression patterns of ELMO1 in 35 MSI-H and 37 MSS CRC tissues (original magnification, × 200, scale bars: 50 μm). Box plot showed statistically significant ELMO1 upregulation in MSI-H CRC samples (*n =* 35) compared with MSS CRC tissues (*n =* 37). (C–E) Kaplan–Meier analysis for the association of ELMO1 expression with overall (upper panel) and CRC-specific survival (lower panel) in the total cohort of 72 cases, MSI-H (*n =* 35) and MSS (*n =* 37) patients (*P < 0.05, **P < 0.01). (C) In the 72-case overall cohort, high ELMO1 expression correlated with shorter overall survival (OS) (*P* = 0.032), but not with DFS. (D) In MSI-H cohort, Kaplan-Meier curves for OS and CRC-specific survival showed that ELMO1-positive patients (*n =* 16) had worse prognosis than ELMO1-negative patients (*n =* 19) (*P* = 0.029, *P* = 0.018). (E) Given the relatively low endogenous ELMO1 expression in MSS CRC patients, we stratified MSS cases into high and low expression subgroups based on the median expression level of ELMO1 in MSS tumors. Further survival analysis showed that ELMO1 expression exerted no significant impact on the prognosis of patients with MSS CRC. (F) MSI-H patients in GEO cohort (*n =* 89) were divided into two groups based on their ELMO1 expression. The heat map of 22 types of immune cells infiltrating in MSI-H CRC tumor tissues. (G) Bar graphs show the expression levels of macrophage markers in patients with high and low ELMO1 expression. The relative expression of CD163 was upregulated in the ELMO1-high group compared with the ELMO1-low group (**P < 0.01). (H) The mRNA expression of CD163 and CD206 was overexpressed in the ELMO1-overexpressing group compared with the ELMO1-low-expressing group (**P < 0.01, ***P < 0.001). MSI-H, microsatellite instability-high; MSS, microsatellite stable; CRC, colorectal cancer; HE, high expression; LE, low expression; NS, no significant.

Transcriptomic profiling was performed on tumor tissues and peripheral blood samples from three MSI-H CRC patients, three MSS CRC patients, and normal colonic tissues from healthy individuals to screen tumor-specific molecular signatures. Three GEO datasets (GSE13067, GSE13294, and GSE13911) were further included for integrative bioinformatic analysis, comprising a total of 89 MSI-H and 140 MSS CRC specimens. Bioinformatic results revealed that ELMO1 was significantly upregulated in MSI-H CRC compared with MSS CRC, indicating a close association between ELMO1 expression and MSI status (Supplementary Fig. S1). To validate these findings, we enrolled 80 fresh tumor tissues (including 21 MSI-H and 59 MSS tumor) for transcriptional analysis and a separate cohort of 72 cases (including 35 MSI-H and 37 MSS tumor) for protein expression (Supplementary Tables S1 and S2). Consistently, ELMO1 expression was markedly increased in MSI-H tumor tissues relative to both MSS tumor tissues and non-tumor tissues (*P *< 0.05, Fig. 1A and B). The correlations between ELMO1 expression and clinicopathological characteristics were subsequently analyzed (Table 1). High ELMO1 expression was significantly correlated with MSI-H status (*P *= 0.017), lymphatic metastasis (*P *= 0.031) and late TNM stages (*P *= 0.025). Then, we further explored the relationships between ELMO1 overexpression and clinicopathological features separately in the MSI-H and MSS cohorts. The clinicopathological analysis indicated that distant metastasis was also associated with ELMO1 expression in MSI-H CRC patients (*P *= 0.003, Table 2). Kaplan-Meier survival analysis showed that ELMO1-positive patients had poorer clinical outcomes than ELMO1-negative patients or the overall cohort of 72 CRC patients (*P *= 0.032, Fig. 1C). Multivariate Cox regression showed that upregulation of ELMO1 was independent prognostic factors in 72 CRC patients (Table 3). Kaplan-Meier survival analysis revealed that MSI-H CRC patients with low ELMO1 expression had markedly longer overall survival and cancer-specific survival than those with high ELMO1 expression (*P *= 0.029 and *P *= 0.018; Fig. 1D and E). Of note, no such significant correlation was detected in the MSS subgroup. Multivariate Cox regression indicated that organ metastasis, TNM stage, and upregulation of ELMO1 were independent prognostic factors in 35 MSI-H CRC patients (Table 4). Taken together, these results highlight that ELMO1 acts as a key regulator in MSI-H CRC.

**Table 2:** The association between ELMO1 expression and clinicopathological features in 35 MSI-H CRCs

Clinicopathological features	Total case	High expression of ELMO1	*P* value
No. of patients	35	16 (45.7%)	
Age (mean 46 years)			0.115
≤46	19	8 (42.1%)	
>46	16	8 (50%)	
Sex			0.433
Male	20	7 (70%)	
Female	15	9 (60%)	
Tumor stage			0.062
pT1–pT2	7	1 (14.3%)	
pT3–pT4	28	15 (53.6%)	
Lymph-node metastasis			0.278
pN0	27	11 (40.7%)	
pN+	8	5 (62.5%)	
Organ metastasis			**0.008**
M0	30	11 (36.7%)	
M1	5	5 (100%)	
TNM stage			0.068
I–II	25	9 (36%)	
III–IV	10	7 (70%)	

Bold values indicate statistically significant differences (*P* < 0.05).

**Table 3:** Univariate and multivariate cox regression analysis in 72 CRCs

Variable	Univariate COX analysis			Multivariate COX analysis		
	HR	95% CI	*P*	HR	95% CI	*P*
Sex (male vs female)	0.45	0.20-0.97	0.042	1.038	0.40–2.69	0.938
Age (≤46 vs >46)	1.49	0.71–3.12	0.29	1.167	0.51–2.68	0.716
MSI status (MSI-H vs MSS)	1.10	0.51–2.36	0.813	0.665	0.22–2.01	0.470
Tumor stage (pT1–pT2 vs pT3–pT4)	0.52	0.17–1.62	0.26	0.320	0.07–1.57	0.160
Lymph-node metastasis (pN0 vs pN+)	3.54	1.63–7.77	**0.001**	2.532	0.32–20.9	0.379
Organ metastasis (M0 vs M1)	4.68	2.10–10.50	**<0.001**	1.888	0.61–2.88	0.273
TNM stage (I–II vs III–IV)	5.14	2.29–11.56	**<0.001**	1.665	0.19–14.40	0.643
ELMO1 expression (low vs high)	2.52	1.16–5.47	**0.019**	3.947	1.19–13.04	**0.024**

Bold values indicate statistically significant differences (*P* < 0.05).

**Table 4:** Univariate and multivariate cox regression analysis in 35 MSI-H CRC cases

Variable	Univariate COX analysis			Multivariate COX analysis		
	HR	95% CI	*P*	HR	95% CI	*P*
Sex (male vs female)	0.878	0.35–2.18	0.78	0.896	0.23–3.51	0.874
Age (≤46 vs >46)	0.58	0.25–1.39	0.225	0.303	0.06–1.67	0.17
Tumor stage (pT1–pT2 vs pT3–pT4)	0.07	0.02–0.32	**<0.001**	0.323	0.04–2.61	0.289
Lymph-node metastasis (pN0 vs pN+)	0.03	0–2.67	0.13	0.316	0.09–1.12	0.074
Organ metastasis (M0 vs M1)	0.04	0–55.49	0.71	0.062	0.18–0.232	**0.025**
TNM stage (I–II vs III–IV)	0.03	0–2.34	0.12	54.867	5.05–595.9	**<0.001**
ELMO1 expression (low vs high)	0.63	0.25–1.54	0.308	6.079	1.03–35.8	**0.046**

Bold values indicate statistically significant differences (*P* < 0.05).

To explore the functional role of ELMO1 in MSI-H CRC, we determined its protein levels in seven CRC cell lines and one normal colonic epithelial cell line by Western blotting (Supplementary Fig. S2A and B). Two shRNAs were employed to silence *ELMO1* in HCT116, DLD-1, SW480, and HT-29 cells (Supplementary Fig. S2C and D). *In vitro* and *in vivo* assays revealed that knockdown of *ELMO1* significantly suppressed cell growth in MSI-H CRC cell lines, HCT116 and DLD-1 (Supplementary Fig. S3A–C). However, ELMO1 downregulation exerted no apparent impact on cell proliferation and colony formation in MSS CRC cell lines, SW480 and HT-29 (Supplementary Fig. S4). Collectively, these findings indicate that elevated ELMO1 expression correlates closely with unfavorable prognosis specifically in patients with MSI-H CRC and facilitates malignant proliferation of MSI-H CRC cells, highlighting the necessity of further exploring the mechanistic role of ELMO1 in driving MSI-H CRC progression.

### ELMO1 recruits M2 macrophages infiltration

**Figure 2:**
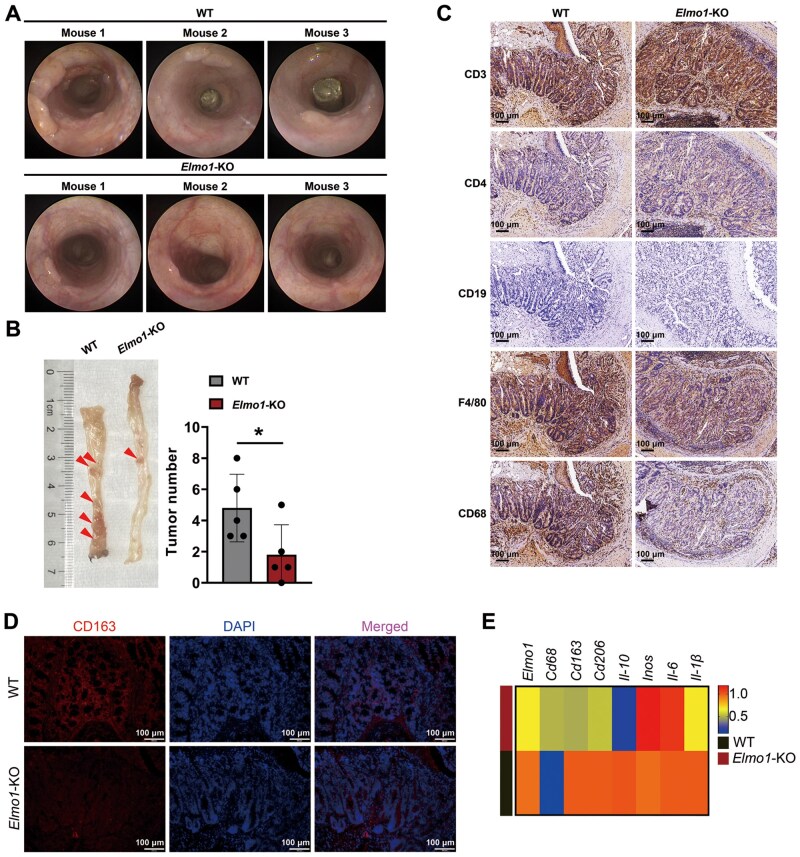
M2 macrophage infiltration in *Elmo1^−/−^* mice models. (A, B) Endoscopic (A) and macroscopic appearance (B) of colon images. The mice were injected with 10 mg/kg AOM and 7 days of 2% DSS administration to establish CAC murine models. Mice were sacrificed on day 84, and colons were obtained. The arrows indicate colon tumors. (C) IHC analysis of immune cell markers expressed on the colonic mucosa in CAC murine model (original magnification, × 200, scale bars: 100 μm). (D) IF analysis of CD163 expressed on the colonic mucosa (original magnification, × 200, scale bars: 100 μm). (E) The relative expression of indicated M1 and M2 macrophages markers in CAC murine models. AOM, azoxymethane; DSS, dextran sodium sulfate; CAC, colitis-associated colorectal cancer; IHC, immunohistochemistry; IF, immunofluorescence.

To explore whether ELMO1 plays a role in the interaction between tumor cells and immune cells in the CRC tumor microenvironment, we quantified the infiltration of 22 immune cell subsets from two GEO datasets using the CIBERSORT algorithm (Fig. 1E). The results showed that the M2 macrophage markers CD206 and CD163, along with the pan-macrophage marker CD68, were significantly elevated in the ELMO1-overexpressed group (*P *< 0.05; Fig. 1G and H). To investigate the relationship between tumor-derived ELMO1 and M2 macrophage infiltration *in vivo*, we generated *Elmo1*-deficient mice (*Elmo1^−/−^*) and established AOM/DSS-induced colitis-associated colorectal cancer (CAC) murine model. Compared with the wild-type mice, *Elmo1^−/−^* mice developed significantly fewer and smaller polypoid lesions, with an approximately two-fold reduction in tumor size (Fig. 2A and B). Immunohistochemistry, immunofluorescence, and qRT-PCR analyses further confirmed that the expression levels of M2 macrophage markers, including CD68, CD206, and CD163, were markedly decreased in CAC tissues from *Elmo1^−/−^* mice relative to wild-type controls (Fig. 2C–E). Collectively, these results demonstrate that tumor-derived ELMO1 facilitates the recruitment of M2 macrophages to tumor sites.

### Extracellular ELMO1 facilitates the recruitment of M2 macrophages

**Figure 3:**
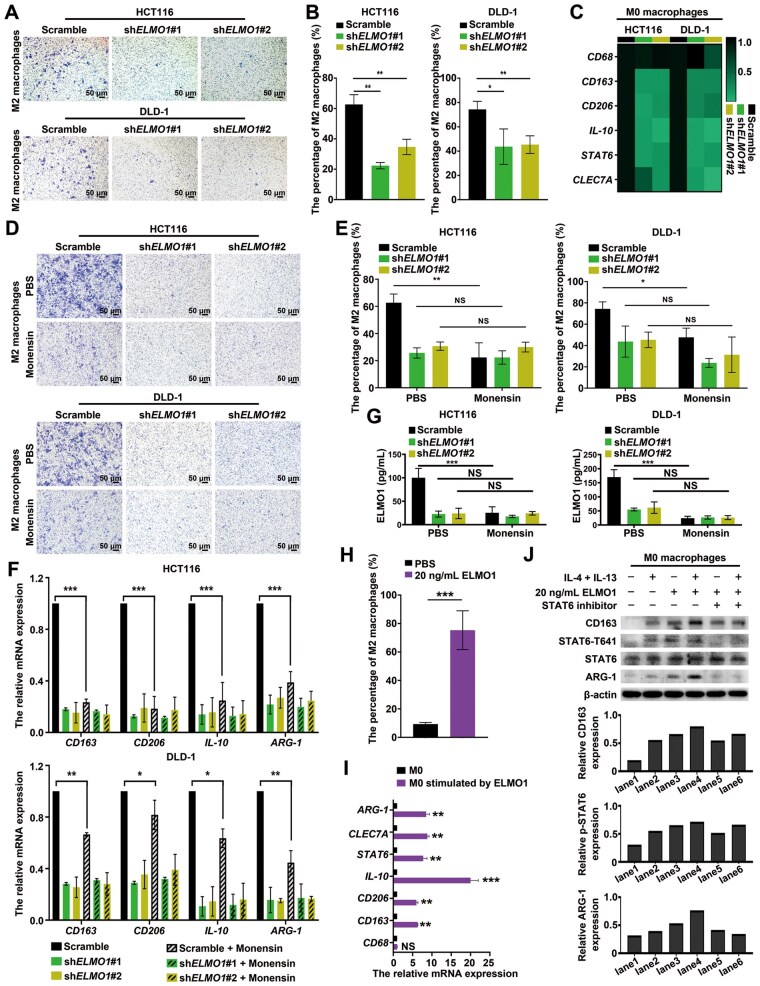
ELMO1 promoted M2 macrophages recruitment and enhanced M0 macrophages differentiation towards M2 macrophages. (A) Representative images and summary of M2 macrophage migration assays induced with *ELMO1*-silenced or scramble tumor cells. Scale bar = 50 μm. (B) Flow cytometric analysis for CD68 and CD163 expression in M0 macrophages after co-culture with scramble tumor cells, sh*ELMO1*#1 tumor cells, and sh*ELMO1*#2 tumor cells. The percentage of M2 macrophages is summarized in the bar chart, and the data were calculated as means ± SEM of three independent experiments (*P < 0.05, **P < 0.01, ****P < 0.0001). (C) Heat map visualization of gene relative expression analyzed from the qRT-PCR results, which illustrated the levels of M2 macrophage markers CD206, CD163, IL-10, CLEC7A, and STAT6, and pan-macrophage marker CD68 in M0 macrophages induced with *ELMO1*-silenced and scramble tumor cells. (D) Representative images and summary of M2 macrophage migration assays induced with ELMO1-silenced or scramble tumor cells under 10 μM monensin treatment. Scale bar = 50 μm. (E) Flow cytometric analysis for CD68 and CD163 expression in M0 macrophages after co-culture with scramble tumor cells, sh*ELMO1*#1 tumor cells, and sh*ELMO1*#2 tumor cells under 10 μM monensin treatment. (F) The mRNA levels of M2 macrophage markers CD206, CD163, IL-10, CCL18, CLEC7A, and STAT6, and pan-macrophage marker CD68 in M0 macrophages induced with *ELMO1*-silenced and scramble tumor cells (*P < 0.05, **P < 0.01, ***P < 0.001). (G) Concentration of ELMO1 in the supernatants of ELMO1-silenced or scramble tumor cells under 10 μM monensin treatment measured by ELISA (***P < 0.001). (H) Flow cytometric analysis for CD68 and CD163 expression in M0 macrophages stimulated with PBS and 20 ng/mL ELMO1 recombinant. The percentage of M2 macrophages is summarized in the bar chart and the data were calculated as means ± SEM of three independent experiments (***P < 0.001). (I) Relative expression of M2 macrophage markers in M0 macrophages and M0 macrophages stimulated with ELMO1 recombinant (100 ng/mL) detected by qPCR (*P < 0.05, **P < 0.01). (J) The expression of CD163, ARG-1 and the phosphorylation levels of STAT6-T641 were evaluated by Western blotting (upper panel). β-actin was used as a loading control. Quantification of protein bands was performed by densitometry using Image J software and data were expressed as the fold change relative to control (lower panel).

Then, we further explored how ELMO1 mediates the crosstalk between tumor cells and M2 macrophages *in vitro*. THP-1 cells were polarized into M0 and M2 macrophages, and a migration assay was subsequently performed. After 48 h of incubation, the number of migrated M2 macrophages was significantly reduced upon induction with ELMO1-silenced tumor cells (Fig. 3A). Furthermore, a co-culture system consisting of M0 macrophages and ELMO1-silenced or scramble control tumor cells was established. Flow cytometry and qRT-PCR results showed that the proportion of CD206+/CD68+ M2 macrophages was notably decreased when M0 macrophages were co-cultured with ELMO1-knockdown tumor cells (Fig. 3B and C; Supplementary Fig. S5A). Monensin, which was applied to modulate exosome-related extracellular cargo release at a high concentration [24], abrogated M2 macrophage infiltration and polarization triggered by shed ELMO1 (Fig. 3D–F and Supplementary Fig. S5B). Enzyme-linked immunosorbent assay (ELISA) assays showed that the expression of ELMO1 decreased remarkably under monensin treatment in MSI-H cancer cells (Fig. 3G). To elucidate the modulatory role of ELMO1 in M2 macrophages, recombinant ELMO1 protein was applied, leading to a notable increase in M2 macrophage migration (Fig. 3H and I; Supplementary Fig. S5C). In addition, Western blot revealed that shed ELMO1 facilitated M2 macrophage polarization specifically by STAT6 activation (Fig. 3K).

**Figure 4:**
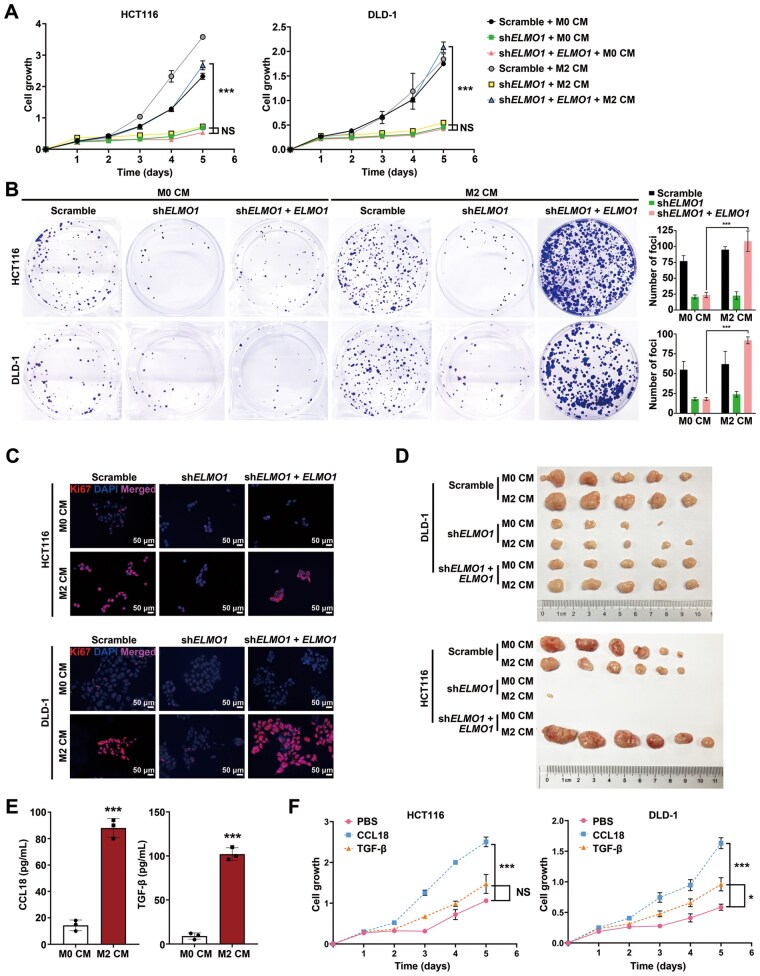
M2 macrophages enhanced the tumor-promoting effects of ELMO1 by CCL18 production. (A) Cell viability of tumor cells detected using the XTT assay after treatment of M2 or M0 CM (***P < 0.001). (B) Foci formation assay of tumor cells was conducted using M2 or M0 CM (***P < 0.001). The numbers of foci were calculated and are shown in the bar chart. (C) Representative images of IF showed the number of Ki67+ tumor cells after treatment of M2 or M0 CM (***P < 0.001). The numbers of Ki67+ tumor cells were calculated and are shown in the bar chart. (D) The tumor volumes of excised tumors from mice injected with tumor cells stimulated by M2 or M0 CM. (E) Concentration of CCL18 and TGF-β in M0 macrophages and M2 macrophages. (F) Cell growth rate was measured by XTT assay with PBS, 20 ng/mL TGF-β or 20 ng/mL CCL18 (*P < 0.05, **P < 0.01). CM, conditioned medium; CCL18, C-C chemokine ligand 18 (CCL18); TGF-β, transforming growth factor-β.

Next, we further explored the effect of M2 macrophages on MSI-H CRC cells. ELMO1-positive (ELMO1(+)) cell lines were constructed by transfecting *ELMO1* cDNA into *ELMO1*-silenced MSI-H CRC cells for subsequent experiments. *In vitro* assays showed that ELMO1(+) cells displayed enhanced proliferation when incubated with M2 macrophage CM (Fig. 4A–C). *In vivo* subcutaneous xenograft results revealed that tumors in the ELMO1(+) group grew markedly larger after treatment with M2 macrophage CM relative to the M0 group (Fig. 4D). Previous studies have reported that M2 macrophages drive tumor initiation and progression via secreting pro-tumor mediators such as CCL18 and TGF-β [25–28]. ELISA and XTT assays verified that M2 macrophages secreted higher levels of CCL18, which prominently promoted the proliferation of ELMO1(+) MSI-H CRC cells (Fig. 4E and F). In summary, tumor cell-derived ELMO1 exosomes induced M2 macrophage polarization and peritumoral accumulation; the recruited M2 macrophages subsequently secrete CCL18, thereby enhancing the growth of ELMO1-overexprssing tumor cells.

### ELMO1 facilitates tumor growth and M2 accumulation via CCL18/PRMT5-dependent methylation

**Figure 5:**
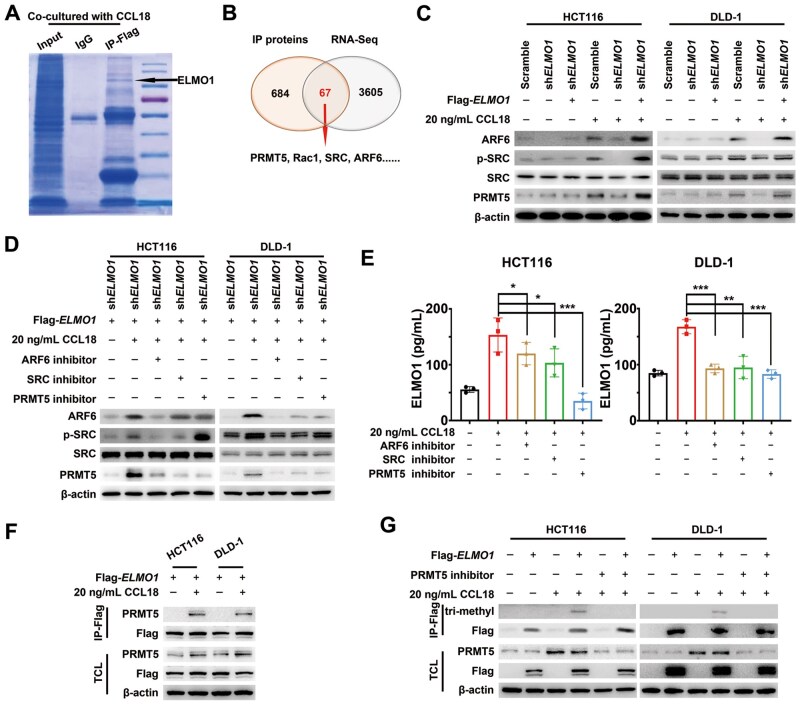
Targeting PRMT5 activation exhibits anti-tumor effects on ELMO1(+) tumor cells. (A) Flag-*ELMO1* was transfected into *ELMO1*-silenced HCT116 cells. Immunoprecipitated flag was used to investigate the interaction protein with ELMO1. Coomassie blue staining of SDS-PAGE gels was used to assess the protein purity and sizes. (B) The Venn plot showed the co-expressed molecules identified via RNA sequencing and IP combined with mass spectrometry. (C) The level of ARF6, PRMT5, SRC, and p-SRC in ELMO1(+) cells with CCL18 was detected by Western blot. (D and E) Cells were treated with 20 μM NAV-2729, 10 nM Dasatinib, and 5 μM GSK591 for 48 h. Western blot showed the level of ARF6, PRMT5, SRC, and p-SRC in ELMO1(+) cells with CCL18 under indicated inhibitions (D). Concentration of ELMO1 in the supernatants of ELMO1(+) tumor cells under indicated treatments measured by ELISA(E). (F) PRMT5/ELMO1 interaction captured by co-immunoprecipitation (Co-IP). The lysate was immunoprecipitated with anti-Flag antibody followed by immunoblotting with anti-PRMT5 antibody in HCT116 and DLD-1 cells. (G) ELMO1(+) cells were treated with 20 ng/mL CCL18 combined with or without GSK591. Immunoprecipitated Flag-ELMO1 was used to evaluate the methylation level.

Based on the above findings, we further explored the molecular mechanism underlying the crosstalk between ELMO1-overexpressing tumor cells and M2 macrophages. Co-IP combined with RNA sequencing was performed to screen key molecular signatures correlated with elevated ELMO1 expression (Fig. 5A and B). We observed that CCL18 treatment markedly upregulated the expression of PRMT5, ARF6, and phosphorylated SRC in ELMO1(+) HCT116 and DLD-1 cells (Fig. 5C). Subsequently, specific pharmacological inhibitors targeting ARF6 (NAV-2729), SRC (Dasatinib), and PRMT5 (GSK591) were applied to verify the changes in the expression of these proteins. Upon inhibitor intervention, the activation of PRMT5, ARF6, and phosphorylated SRC was obviously attenuated in ELMO1(+) MSI-H CRC cells, whereas the total SRC expression remained unaltered (Fig. 5D). Notably, ELISA results showed that GSK591 significantly reduced extracellular ELMO1 secretion compared with the other two inhibitors, indicating that PRMT5 functions as a specific upstream regulator of ELMO1 (Fig. 5E). Co-IP assay confirmed a direct interaction between PRMT5 and ELMO1 (Fig. 5F). In GSK591-treated MSI-H CRC cells, endogenous ELMO1 methylation levels were remarkably diminished (Fig. 5G). Collectively, these results indicated that CCL18 secreted from M2 macrophages triggered PRMT5 directly interacted with ELMO1 and modulated the methylation modification of ELMO1.

**Figure 6:**
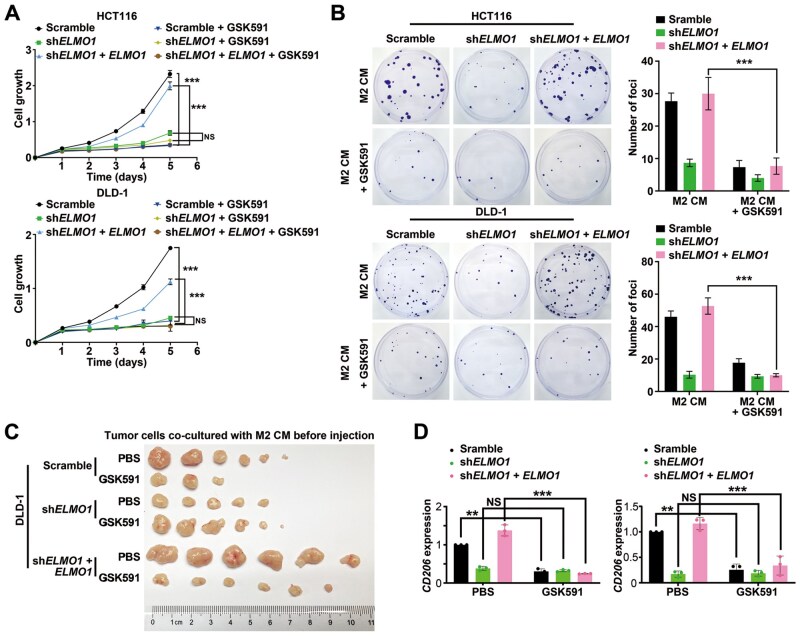
CCL18/PRMT5/ELMO1 axis promotes MSI-H cancer cell growth. (A) Cell viability of tumor cells detected with the XTT assay after treatment of M2 CM combined with PBS or GSK591 (*P < 0.05, **P < 0.01, ***P < 0.001). (B) Foci formation assay of tumor cells was conducted using M2 CM with/without PBS or GSK591. (C) Representative images of the xenograft tumors formed in nude mice. (D) Relative expression of M2 macrophage markers CD206 detected using qRT-PCR (*P < 0.05, **P < 0.01, ***P < 0.001). CM, conditioned medium.

We next investigated whether pharmacological blockade of PRMT5 affects the malignant progression of ELMO1 (+) MSI-H tumor cells. XTT and colony formation assays revealed that PRMT5 inhibition markedly suppressed the proliferation of ELMO1-positive MSI-H CRC cells (Fig. 6A and B). Moreover, the anti-tumor effect of GSK591 was further validated *in vivo* using xenograft tumor models. GSK591 treatment significantly reduced xenograft tumor growth and volume (Fig. 6C). Tumor tissues were harvested for subsequent mechanistic validation of the PRMT5 inhibitor *in vivo*. qRT-PCR results showed that the M2 macrophage markers CD206 was significantly downregulated in the ELMO1-positive group following GSK591 treatment relative to the PBS control group (Fig. 6D). These findings suggest that targeting PRMT5 may serve as a promising therapeutic strategy for the treatment of ELMO1-positive MSI-H CRC.

### ELMO1 protein methylation is a potential therapeutic target for CRC immunotherapy

**Figure 7:**
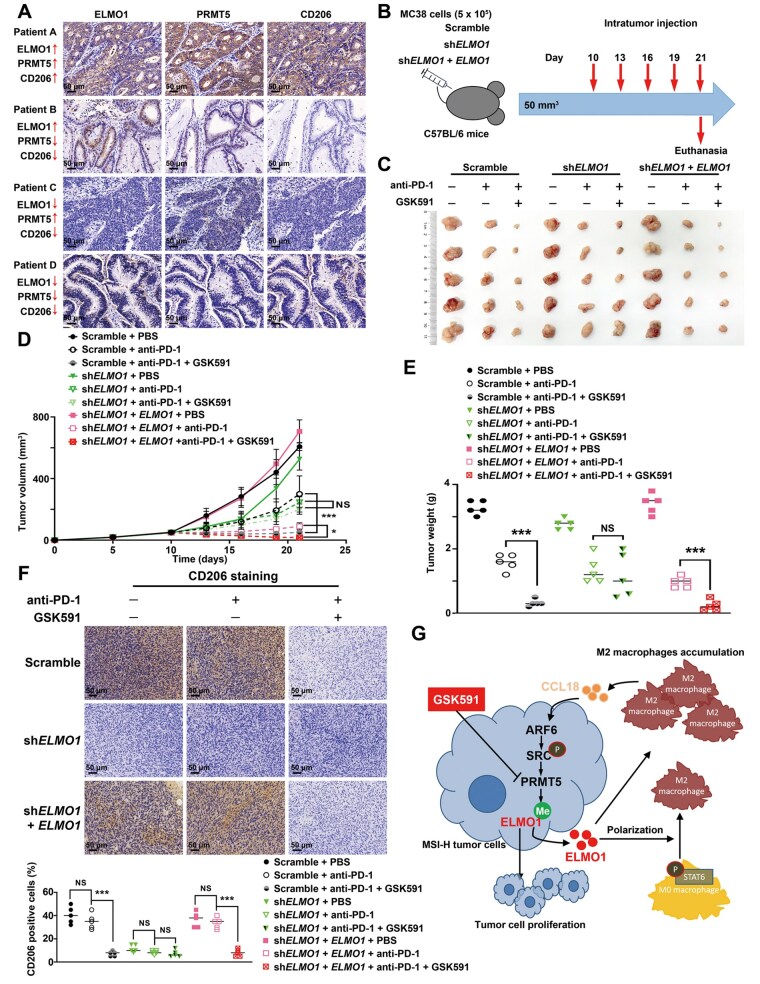
PRMT5 blockade targeting ELMO1 efficiently improves the anticancer activity of anti-PD-1 antibody. (A) Immunohistochemical staining of ELMO1, PRMT5, and CD206 in MSI-H CRC specimens. Representative images showed ELMO1, PRMT5, and CD206 expression patterns in different CRC cases. Patient A: positive for ELMO1, PRMT5, and CD206. Patient B: low ELMO1 with negative PRMT5 and CD206 staining. Patient C: low PRMT5 with negative ELMO1 and CD206 staining. Patient D: negative for ELMO1, PRMT5, and CD206 (original magnification, ×200, scale bars: 50μm). (B) Design for establishment of MC38 syngeneic tumors and treatment with PBS, anti-PD-1, or combination of GSK591 and anti-PD-1 antibody in C57BL/6 mice. (C) Representative image of xenografts in different groups. (D, E) Tumor volume (D) and weight (E) from tumors in (C). (F) CD206 expression was measured by IHC in these tumors from (C). Scale bar = 50 μm (G) Graphical outline of the proposed mechanism in this study. IHC, immunohistochemistry.

To explore whether ELMO1 participates in the regulation of anti-tumor immunity, we detected the expression of ELMO1, PRMT5, and CD206 in pre-immunotherapy archived specimens from 35 MSI-H CRC patients receiving subsequent anti-PD-1/PD-L1 treatment (nivolumab, pembrolizumab, or atezolizumab), and further analyzed the correlation between marker expression and clinical therapeutic outcomes (Supplementary Table S2). In anti-PD-1-resistant cases (exemplified by patient A), all three markers were highly expressed. By contrast, none of the three proteins showed positive immunohistochemical staining in anti-PD-1-sensitive cases (exemplified by patient D) (Fig. 7A). In specimens with undetectable or marginal PRMT5 expression (exemplified by patient B and patient C), only sparse infiltration of the M2 macrophage marker CD206 was observed. The prominent positive correlation of CD206 staining with ELMO1 and PRMT5 indicated that high tumoral ELMO1 and PRMT5 expression is closely associated with anti-PD-1 therapy resistance.

Given that reduced infiltration of M2-like TAMs has been reported to correlate with improved immunotherapeutic efficacy in multiple malignancies [29], we further explored whether PRMT5 blockade could potentiate anti-PD-1 immunotherapy in ELMO1-upregulated CRC. As expected, pharmacological inhibition of PRMT5 markedly enhanced the therapeutic efficacy of anti-PD-1 treatment and suppressed tumor growth in MC38 (MSI-H) syngeneic tumor models (Fig. 7B–E). Immunohistochemistry staining further confirmed that combined treatment with PRMT5 inhibitor and anti-PD-1 antibody remarkably reduced the accumulation of M2 macrophages within tumor tissues (Fig. 7F).

These results indicated that blockade of the PRMT5/ELMO1 axis is a promising feasible therapeutic strategy for MSI-H CRC patients with high ELMO1 expression.

## Discussion

In this study, we delineated the mechanistic underpinnings linking elevated ELMO1 expression to dismal clinical prognosis in CRC patients and validated ELMO1 as a promising novel biomarker for precise subtyping of the MSI-H subtype.

Evidence has indicated the positive effects of ELMO1 on promoting cancer cell proliferation, invasion, and metastasis [17, 30, 31]. To dissect the clinical implication of ELMO1 in CRC pathogenesis, we first profiled its expression in clinical CRC specimens. Consistently with a prior clinical investigation [15], ELMO1 was significantly overexpressed in tumor tissues relative to adjacent normal tissues. Intriguingly, the expression of ELMO1 was specifically upregulated in MSI-H CRC patients and closely correlated with unfavorable survival outcomes. *In vitro* functional assays showed that ELMO1 selectively facilitated the growth of MSI-H CRC cells (HCT116 and DLD-1), whereas it exerted negligible effects on the proliferative phenotype of MSS CRC cells (HT29 and SW480). Further bioinformatic analyses revealed that MSI-H patients with high ELMO1 expression exhibited prominent M2 macrophage infiltration. Previous studies have implicated ELMO1 in modulating tumor immune microenvironment and malignant progression. For instance, ELMO1 drives macrophage cytoskeleton rearrangement via activating Rac-1 signaling to facilitate lung cancer metastasis [32]. In gastric cancer, ELMO1 interacts with Med31 to potentiate DOCK180 activity, thereby triggering EMT and M2 macrophage polarization upon *Helicobacter pylori* infection [33]. Consistent with these prior observations, we hypothesized that ELMO1 in MSI-H CRC might also interact with macrophages via a transcriptional network. Co-culture and migration assays further confirmed that ELMO1-positive MSI-H CRC cells efficiently drove M0 macrophage polarization toward the M2 phenotype and induced the accumulation of M2 macrophages; conversely, *ELMO1* knockdown largely abrogated these pro-tumor immune phenotypes.

Oncogenic activation in tumor cells profoundly promotes the biogenesis and release of tumor-derived extracellular vesicles (EVs). Tumor-derived EVs are loaded with abundant functional molecules, including complement components, transcription factors, and membrane proteins, which can be internalized by immune cells such as macrophages, neutrophils, and myeloid-derived suppressor cells [34]. Upon EV uptake, recipient immune cells activate downstream signaling cascades and secrete multiple chemokines and inflammatory cytokines, further recruiting circulating immune cells to tumor sites. This EV-mediated intercellular crosstalk reshapes immune cell infiltration, remodels the tumor microenvironment, and ultimately facilitates tumor immune escape, malignant progression, and distant metastasis [35]. Dual bioinformatic prediction with SignalP 6.0 and TMHMM 2.0 demonstrated that ELMO1 had no classical N-terminal signal peptide sequence, suggesting that it is not secreted via the classical ER/Golgi secretory pathway. Based on this framework, we pharmacologically blocked EV secretion in CRC cells using high-dose monensin intervention [24]. Results showed that the recruitment of M2 macrophages toward ELMO1-positive tumor cells was significantly attenuated under monensin treatment, accompanied by a remarkable decline in the proportion of M0-to-M2 phenotypic transformation. Our experimental data showed that monensin-mediated EV inhibition markedly reduced the abundance of secreted ELMO1 in tumor cell supernatant. Then, exogenous supplementation of recombinant ELMO1 protein was sufficient to drive M0 macrophage polarization toward the M2 phenotype. Collectively, these observations corroborate that tumor cell-secreted ELMO1 functions as a key paracrine factor to modulate M2 macrophage migration and polarization. Study reported that M2 macrophages promote tumor initiation and progression by secreting pro-tumor mediators such as CCL18, TGF-β, and IL-10 [25–28]. In our study, CCL18 secreted by M2 macrophages upregulated ARF6 and phosphorylated SRC and PRMT5 in ELMO1-positive MSI-H cancer cells. Treatment with NAV-2729, Dasatinib, or GSK591, combined with M2 macrophage CM or recombinant CCL18, markedly reduced the level of extracellular ELMO1 in MSI-H tumor cells compared with PBS control. Meanwhile, GSK591 treatment significantly decreased the methylation level of ELMO1, thereby reducing extracellular ELMO1 release. Moreover, we explored the potential value of PRMT5 inhibitors in modulating the proliferation of ELMO1-positive MSI-H CRC cells. The PRMT5 inhibitor GSK591 obviously suppressed cell viability *in vitro* and inhibited tumor growth *in vivo* in ELMO1-positive MSI-H models relative to the untreated group. Collectively, these findings highlight the great prospect of PRMT5-targeted combination therapy for ELMO1-high MSI-H CRC.

Nevertheless, several inherent limitations of the present study should be acknowledged and addressed in future investigations. First, the precise methylation sites of ELMO1 and the detailed mechanism underlying this feedback regulatory cascade have not been fully clarified, which deserves in-depth exploration in future investigations. Second, the precise sorting mechanism of ELMO1 into specific EV subtypes (e.g. exosomes) and its detailed cargo function in regulating M2 macrophage behaviors remain to be further characterized, which will be prioritized in our subsequent mechanistic research. Further validation using *in vivo* animal models and large-scale clinical cohorts is therefore warranted to consolidate our current findings.

In summary, our study uncovers a previously unrecognized ELMO1 methylation-mediated crosstalk axis between MSI-H CRC cells and M2 macrophages, which governs immunotherapy resistance and tumor progression. Targeting PRMT5-dependent ELMO1 methylation provides a novel strategy to remodel the immunosuppressive TME and restore immunotherapy responsiveness. Collectively, ELMO1 represents both a robust prognostic biomarker and a promising druggable target for overcoming anti-PD-1 resistance in MSI-H CRC patients.

## Supplementary Material

goag056_Supplementary_Data

## References

[1] Sung H , Ferlay J, Siegel RL et al Global cancer statistics 2020: GLOBOCAN estimates of incidence and mortality worldwide for 36 cancers in 185 countries. CA Cancer J Clin 2021;71:209–49. 33538338 10.3322/caac.21660

[2] Zaanan A , Shi Q, Taieb J et al Role of deficient DNA mismatch repair status in patients with stage III colon cancer treated with FOLFOX adjuvant chemotherapy a pooled analysis from 2 randomized clinical trials. JAMA Oncol 2018;4:379–83. 28983557 10.1001/jamaoncol.2017.2899 PMC5784452

[3] Vilar E , Gruber SB. Microsatellite instability in colorectal cancer-the stable evidence. Nat Rev Clin Oncol 2010;7:153–62. 20142816 10.1038/nrclinonc.2009.237 PMC3427139

[4] Taieb J , Svrcek M, Cohen R et al Deficient mismatch repair/microsatellite unstable colorectal cancer: diagnosis, prognosis and treatment. Eur J Cancer 2022;175:136–57. 36115290 10.1016/j.ejca.2022.07.020

[5] Le DT , Durham JN, Smith KN et al Mismatch repair deficiency predicts response of solid tumors to PD-1 blockade. Science 2017;357:409–13. 28596308 10.1126/science.aan6733 PMC5576142

[6] Andre T , Shiu KK, Kim TW et al; KEYNOTE-177 Investigators. Pembrolizumab in microsatellite-instability-high advanced colorectal cancer. N Engl J Med 2020;383:2207–18. 33264544 10.1056/NEJMoa2017699

[7] Bao Y , Zhai J, Chen H et al Targeting m(6)A reader YTHDF1 augments antitumour immunity and boosts anti-PD-1 efficacy in colorectal cancer. Gut 2023;72:1497–509. 36717220 10.1136/gutjnl-2022-328845 PMC10359538

[8] Cassetta L , Pollard JW. Targeting macrophages: therapeutic approaches in cancer. Nat Rev Drug Discov 2018;17:887–904. 30361552 10.1038/nrd.2018.169

[9] Komohara Y , Fujiwara Y, Ohnishi K et al Tumor-associated macrophages: potential therapeutic targets for anti-cancer therapy. Adv Drug Deliv Rev 2016;99:180–5. 26621196 10.1016/j.addr.2015.11.009

[10] Jones KI , Tiersma J, Yuzhalin AE et al Radiation combined with macrophage depletion promotes adaptive immunity and potentiates checkpoint blockade. EMBO Mol Med 2018;10:e9342. 30442705 10.15252/emmm.201809342 PMC6284388

[11] Wu Q , Zhou W, Yin S et al Blocking triggering receptor expressed on myeloid cells-1-positive tumor-associated macrophages induced by hypoxia reverses immunosuppression and anti-programmed cell death ligand 1 resistance in liver cancer. Hepatology 2019;70:198–214. 30810243 10.1002/hep.30593 PMC6618281

[12] Xiao Z , Nie K, Han T et al Development and validation of a TNF family-based signature for predicting prognosis, tumor immune characteristics, and immunotherapy response in colorectal cancer patients. J Immunol Res 2021;2021:6439975. 34541005 10.1155/2021/6439975 PMC8448595

[13] Sarkar A , Tindle C, Pranadinata RF et al ELMO1 regulates autophagy induction and bacterial clearance during enteric infection. J Infect Dis 2017;216:1655–66. 29029244 10.1093/infdis/jix528 PMC5853658

[14] Morioka S , Kajioka D, Yamaoka Y et al Chimeric efferocytic receptors improve apoptotic cell clearance and alleviate inflammation. Cell 2022;185:4887–903.e17. 36563662 10.1016/j.cell.2022.11.029 PMC9930200

[15] Wen B , Li S, Ruan L et al Engulfment and cell motility protein 1 fosters reprogramming of tumor-associated macrophages in colorectal cancer. Cancer Sci 2023;114:410–22. 36310143 10.1111/cas.15628 PMC9899619

[16] Das S , Sarkar A, Choudhury SS et al ELMO1 has an essential role in the internalization of Salmonella typhimurium into enteric macrophages that impacts disease outcome. Cell Mol Gastroenterol Hepatol 2015;1:311–24. 26878033 10.1016/j.jcmgh.2015.02.003 PMC4747049

[17] Tocci S , Ibeawuchi SR, Das S et al Role of ELMO1 in inflammation and cancer-clinical implications. Cell Oncol (Dordr) 2022;45:505–25. 35668246 10.1007/s13402-022-00680-x PMC12978106

[18] Peng C , Zhao G, Pei B et al A novel plasma-based methylation panel for upper gastrointestinal cancer early detection. Cancers (Basel) 2022;14:5282. 36358701 10.3390/cancers14215282 PMC9656240

[19] Xue Y , Zhao G, Song L et al The signature of cancer methylation markers in maternal plasma: factors influencing the development and application of cancer liquid biopsy assay. Gene 2024;906:148261. 38342253 10.1016/j.gene.2024.148261

[20] Park YL , Cho SB, Park SY et al Engulfment and cell motility 1 (ELMO1) regulates tumor cell behavior and predicts prognosis in colorectal cancer. Anticancer Res 2022;42:5343–55. 36288887 10.21873/anticanres.16058

[21] Xu X , Yin F, Guo M et al Quantitative proteomic analysis of exosomes from umbilical cord mesenchymal stem cells and rat bone marrow stem cells. Proteomics 2023;23:e2200204. 36408942 10.1002/pmic.202200204

[22] Hurwitz SN , Rider MA, Bundy JL et al Proteomic profiling of NCI-60 extracellular vesicles uncovers common protein cargo and cancer type-specific biomarkers. Oncotarget 2016;7:86999–7015. 27894104 10.18632/oncotarget.13569 PMC5341331

[23] Newman AM , Liu CL, Green MR et al Robust enumeration of cell subsets from tissue expression profiles. Nat Methods 2015;12:453–7. 25822800 10.1038/nmeth.3337 PMC4739640

[24] Payne LG , Kristensson K. The effect of cytochalasin D and monensin on enveloped vaccinia virus release. Arch Virol 1982;74:11–20. 6891579 10.1007/BF01320778

[25] Nguyen TN , Nguyen-Tran HH, Chen CY et al IL6 and CCL18 mediate cross-talk between VHL-deficient kidney cells and macrophages during development of renal cell carcinoma. Cancer Res 2022;82:2716–33. 35666812 10.1158/0008-5472.CAN-21-3749 PMC9662868

[26] Long L , Hu Y, Long T et al Tumor-associated macrophages induced spheroid formation by CCL18-ZEB1-M-CSF feedback loop to promote transcoelomic metastasis of ovarian cancer. J Immunother Cancer 2021;9:e003973. 34969774 10.1136/jitc-2021-003973 PMC8718465

[27] Murai H , Kodama T, Maesaka K et al Multiomics identifies the link between intratumor steatosis and the exhausted tumor immune microenvironment in hepatocellular carcinoma. Hepatology 2023;77:77–91. 35567547 10.1002/hep.32573 PMC9970024

[28] Zhu X , Liang R, Lan T et al Tumor-associated macrophage-specific CD155 contributes to M2-phenotype transition, immunosuppression, and tumor progression in colorectal cancer. J Immunother Cancer 2022;10:e004219. 36104099 10.1136/jitc-2021-004219 PMC9476138

[29] Zhou C , Weng J, Liu C et al Disruption of SLFN11 deficiency-induced CCL2 signaling and macrophage M2 polarization potentiates anti-PD-1 therapy efficacy in hepatocellular carcinoma. Gastroenterology 2023;164:1261–78. 36863689 10.1053/j.gastro.2023.02.005

[30] Luo T , Chen M, Zhao Y et al Macrophage-associated lncRNA ELMO1-AS1: a novel therapeutic target and prognostic biomarker for hepatocellular carcinoma. Onco Targets Ther 2019;12:6203–16. 31498334 10.2147/OTT.S213833 PMC6689543

[31] Park YL , Choi JH, Park SY et al Engulfment and cell motility 1 promotes tumor progression via the modulation of tumor cell survival in gastric cancer. Am J Transl Res 2020;12:7797–811. 33437361 PMC7791502

[32] Zhang Y , Wang X, Gu Y et al Complement C3 of tumor-derived extracellular vesicles promotes metastasis of RCC via recruitment of immunosuppressive myeloid cells. Proc Natl Acad Sci USA 2025;122:e2420005122. 39847320 10.1073/pnas.2420005122 PMC11789090

[33] Lu T , Yu T, He C et al Interaction between ELMO1 DNA methylation and Med31 promotes H. pylori-induced gastric cancer EMT and intestinal metaplasia via M2 polarization. Sci Rep 2026;16:5201. 41535333 10.1038/s41598-026-35314-x PMC12881572

[34] Belgrader P , Young S, Yuan B et al A battery-powered notebook thermal cycler for rapid multiplex real-time PCR analysis. Anal Chem 2001;73:286–9. 11199979 10.1021/ac000905v

[35] Rubenich DS , Domagalski JL, Gentil GFS et al The immunomodulatory ballet of tumour-derived extracellular vesicles and neutrophils orchestrating the dynamic CD73/PD-L1 pathway in cancer. J Extracell Vesicles 2024;13:e12480. 38978304 10.1002/jev2.12480 PMC11231043

