# Supplementary material for: Targeting engulfment and cell motility 1 protein methylation attenuates M2 macrophage infiltration and boosts anti-PD-1 efficacy in colorectal cancer
Source: Gastroenterol Rep (Oxf). 2026 Jun 19;14:goag056. doi: 10.1093/gastro/goag056 (PMC13282086; doi:10.1093/gastro/goag056)
Supplement: goag056_Supplementary_Data [file goag056_supplementary_data.docx]

**Supplementary Information**

**Supplementary Figure 1. Differentially expressed genes between MSI-H and MSS samples.**

**
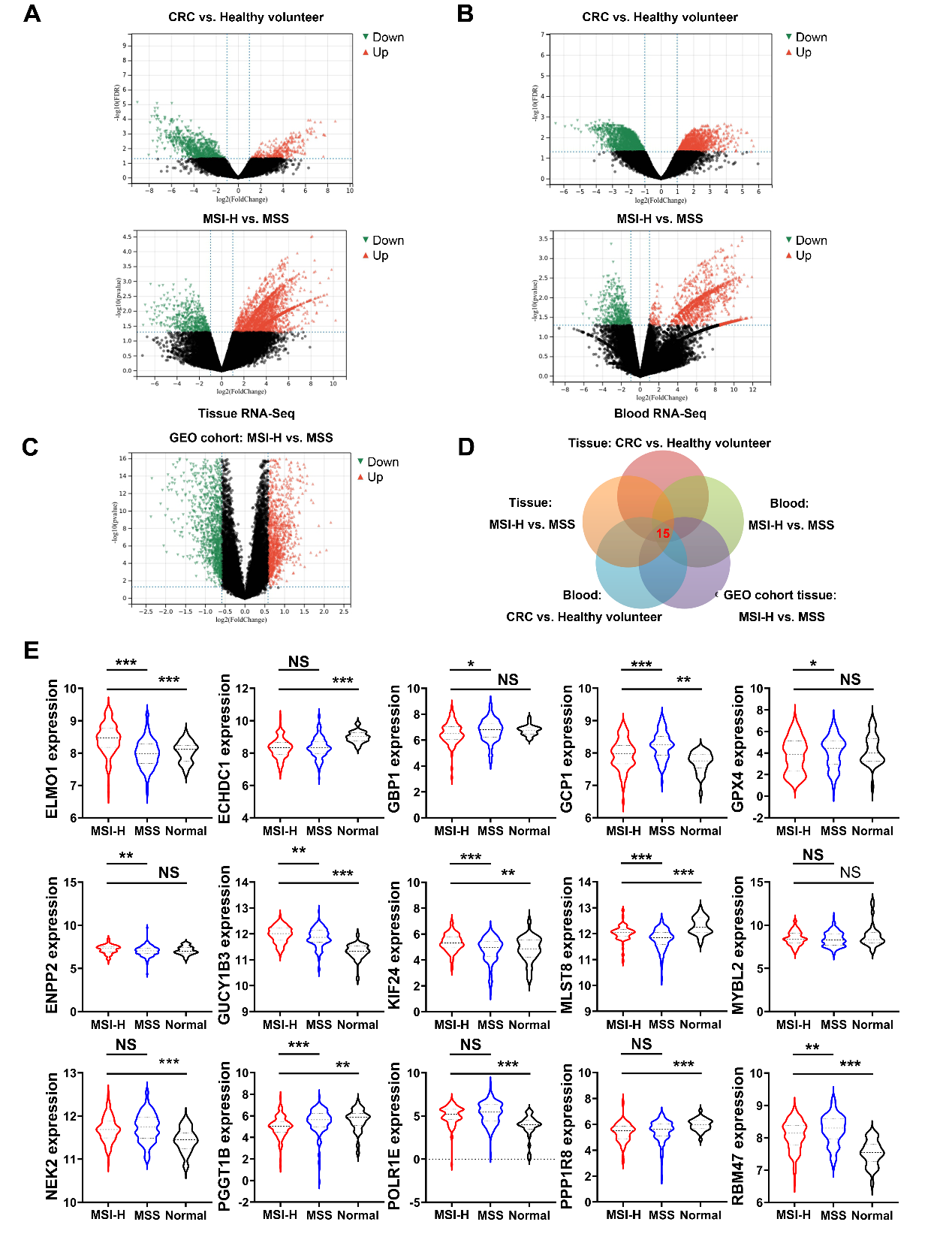
**

(A-B) Volcano plot showing DEGs in CRC tissue (A) and peripheral blood (B). (C)Volcano plot showing DEGs in GEO cohort. (D) Venn plot showing the co-expressional genes. (E) Expression of 15 genes in (D) between MSI-H, MSS and normal tissues (* *P* < 0.05, ** *P* < 0.01, *** *P* < 0.001). DEGs-differentially expressed genes; GEO-gene expression omnibus; CRC-colorectal cancer; MSI-H-microsatellite instability-high; MSS-microsatellite stable; NS-no significance

**Supplementary Figure 2. Establishment of *ELMO1*-silenced cells.**


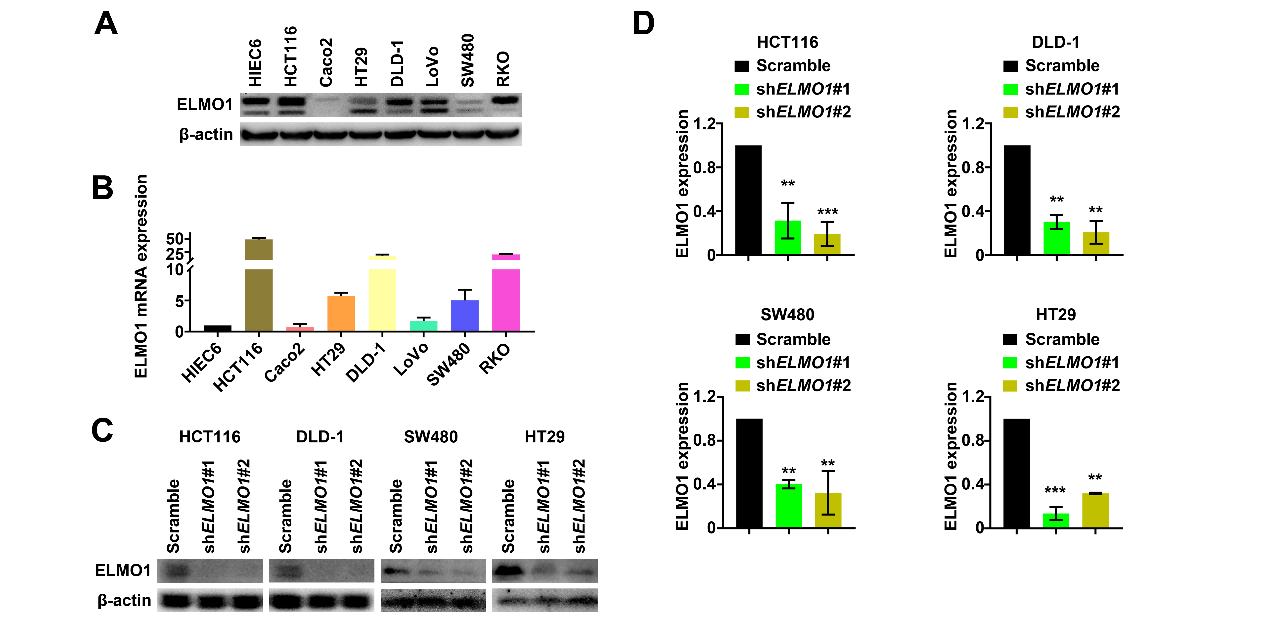


(A-B) Western blot and qPCR analysis of expression of ELMO1 in 7 CRC cell lines and 1 normal colonic epithelial cell line. (C-D) HCT116, DLD-1, SW480 and HT-29 cells were stably transfected with shRNAs against ELMO1 or scramble shRNA as control, respectively. The expression of ELMO1 was detected by Western blot analysis, and β-actin was used as a loading control(C). *ELMO1* mRNA level was detected in *ELMO1*-silenced and control cells by qRT-PCR(D) (** *P* < 0.01, *** *P* < 0.001). ELMO1- Engulfment and cell motility protein 1; qRT-PCR- quantitative real-time polymerase chain reaction

**Supplementary Figure 3. Knockdown of ELMO1 suppressed MSI CRC cancer cell growth.**


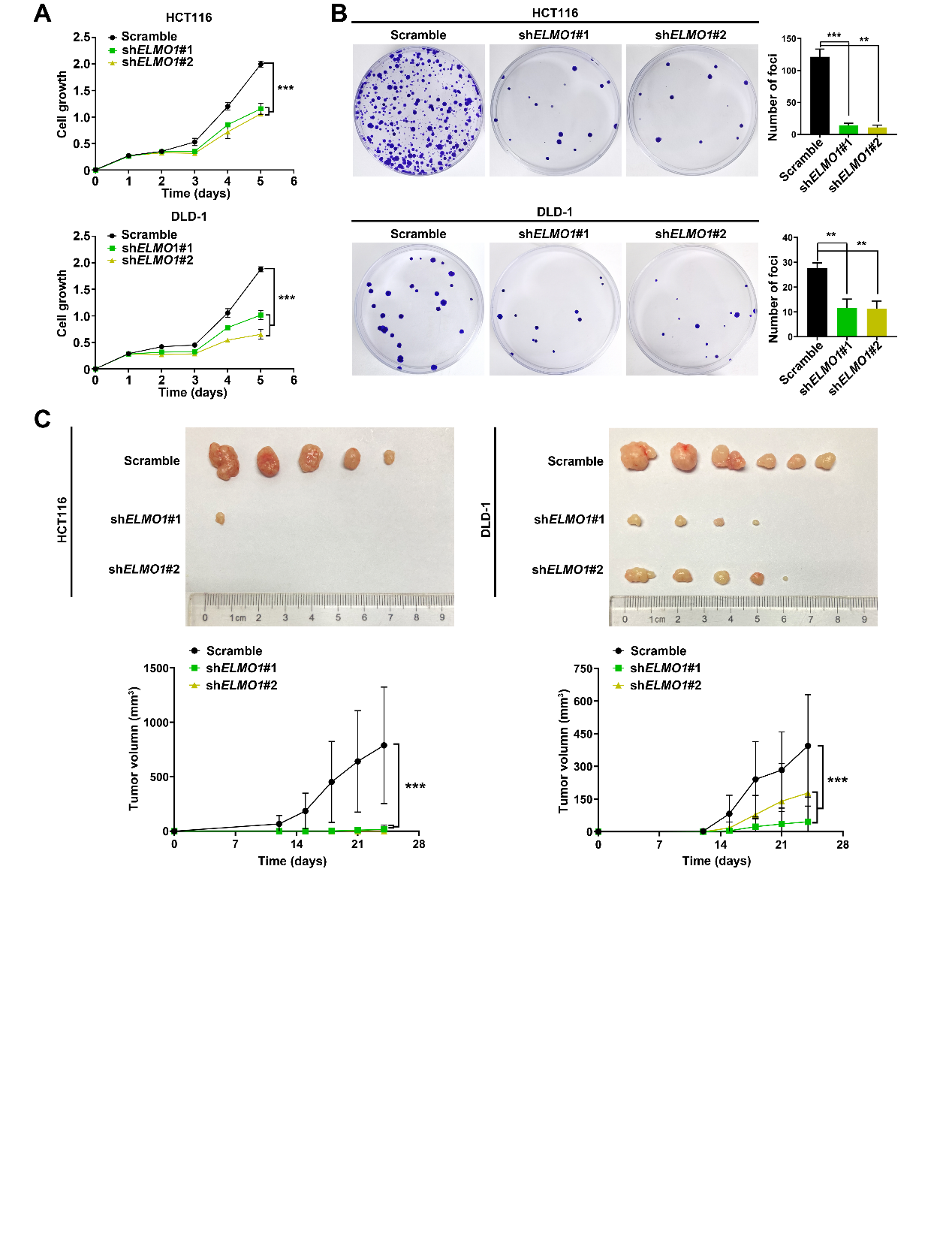


(A) XTT assay showed that ablation of endogenous ELMO1 inhibited cell proliferation in HCT116 and DLD-1 cells. The results were expressed as the mean ± SD of three independent experiments. (*** *P* < 0.001) (B) Foci formation assay indicated that silencing of ELMO1 inhibited cell colony formation. Quantitative analyses of foci numbers were shown in the right panel. Values were reflected as the mean ± SD of three independent experiments. (*** *P*< 0.001) (C) Representative images of the xenograft tumors formed in nude mice. Growth curves of tumors derived from the indicated cell lines are shown (*** *P* < 0.001).

**Supplementary Figure 4. ELMO1 had no effect on MSS CRC cells.**


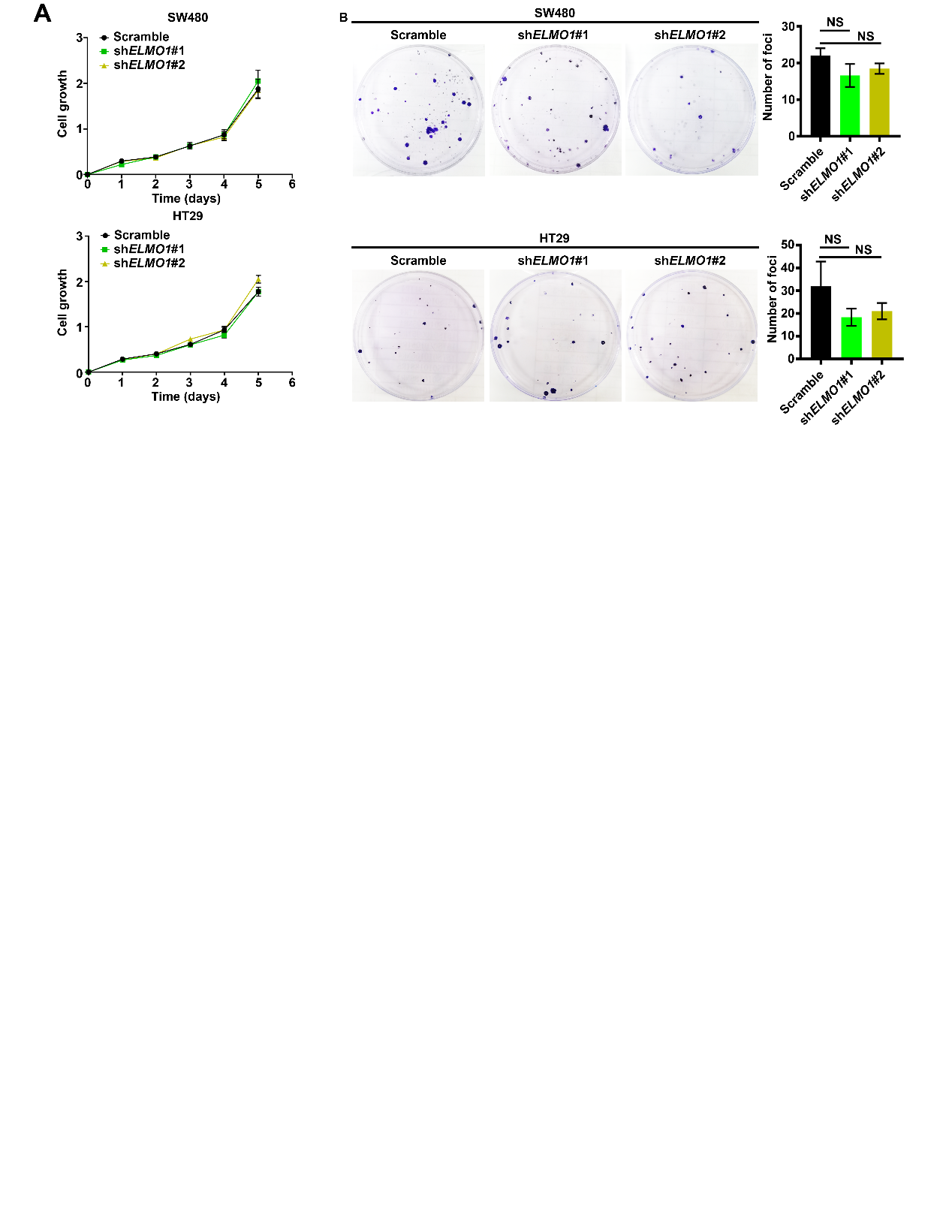


(A) Cell growth rate was measured by XTT assay. (B) Foci formation assay indicated that silencing of ELMO1 did not inhibit cell colony formation.

**Supplementary Figure 5. The phenotypes of ELMO1-induced macrophages.**

**
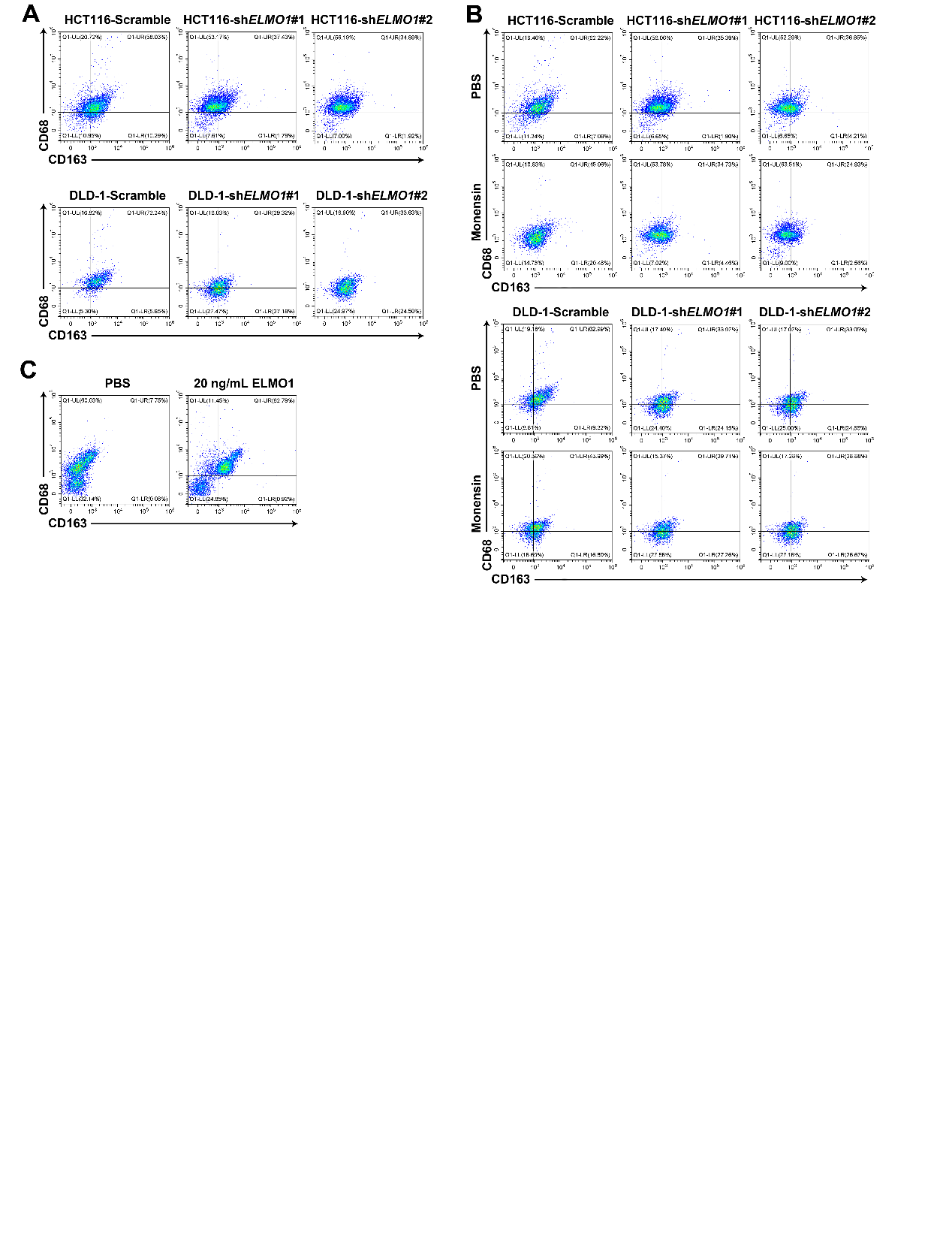
**

(A) Flow cytometric images of CD68 and CD163 expression in M0 macrophages after co-culture with *ELMO1*-silenced or scramble HCT116 and DLD-1 cells. (B) Flow cytometric images of CD68 and CD163 expression in M0 macrophages after co-culture with *ELMO1*-silenced or scramble cancer cells after blocking with 10 μM monensin. (C) Flow cytometric analysis for CD68 and CD163 expression in M0 macrophages stimulated with PBS and 20 ng/mL ELMO1 recombinant.

**Supplementary Table 1. Patients’ clinicopathological features in 80 colorectal cancer informative cases.**

| ID | Sex | Age | T stage | N stage | M stage | TNM stage | MSI status |
| --- | --- | --- | --- | --- | --- | --- | --- |
| 1 | Male | 31 | 3 | 0 | 0 | 2 | MSI |
| 2 | Male | 44 | 4 | 2 | 0 | 3 | MSS |
| 3 | Female | 50 | 3 | 0 | 0 | 2 | MSS |
| 4 | Female | 51 | 3 | 1 | 0 | 3 | MSS |
| 5 | Female | 55 | 3 | 0 | 0 | 2 | MSS |
| 6 | Male | 55 | 4 | 1 | 0 | 3 | MSS |
| 7 | Female | 56 | 3 | 0 | 0 | 2 | MSS |
| 8 | Female | 56 | 3 | 0 | 1 | 4 | MSS |
| 9 | Female | 57 | 3 | 1 | 0 | 3 | MSS |
| 10 | Male | 59 | 3 | 2 | 0 | 3 | MSS |
| 11 | Male | 60 | 3 | 1 | 0 | 3 | MSS |
| 12 | Male | 60 | 3 | 2 | 0 | 3 | MSS |
| 13 | Female | 63 | 3 | 0 | 0 | 2 | MSS |
| 14 | Male | 64 | 3 | 2 | 0 | 3 | MSS |
| 15 | Male | 67 | 4 | 2 | 0 | 3 | MSS |
| 16 | Female | 72 | 3 | 0 | 0 | 2 | MSS |
| 17 | Female | 74 | 3 | 0 | 0 | 2 | MSS |
| 18 | Male | 76 | 4 | 0 | 0 | 2 | MSS |
| 19 | Female | 76 | 3 | 0 | 0 | 2 | MSS |
| 20 | Male | 84 | 4 | 2 | 0 | 3 | MSS |
| 21 | Female | 39 | 1 | 0 | 0 | 1 | MSS |
| 22 | Female | 55 | 1 | 1 | 0 | 3 | MSS |
| 23 | Female | 57 | 1 | 2 | 0 | 3 | MSS |
| 24 | Male | 61 | 1 | 1 | 0 | 3 | MSS |
| 25 | Female | 74 | 1 | 1 | 0 | 3 | MSS |
| 26 | Male | 36 | 1 | 1 | 0 | 3 | MSS |
| 27 | Female | 38 | 1 | 1 | 0 | 3 | MSS |
| 28 | Male | 60 | 1 | 1 | 0 | 3 | MSS |
| 29 | Male | 53 | 1 | 1 | 0 | 3 | MSS |
| 30 | Male | 63 | 1 | 0 | 0 | 1 | MSS |
| 31 | Male | 69 | 1 | 1 | 0 | 3 | MSS |
| 32 | Female | 54 | 1 | 0 | 0 | 1 | MSS |
| 33 | Female | 52 | 1 | 1 | 0 | 3 | MSS |
| 34 | Male | 67 | 1 | 1 | 0 | 3 | MSS |
| 35 | Female | 61 | 1 | 1 | 0 | 3 | MSS |
| 36 | Male | 37 | 1 | 0 | 0 | 1 | MSS |
| 37 | Female | 48 | 1 | 1 | 0 | 3 | MSS |
| 38 | Female | 63 | 1 | 2 | 0 | 3 | MSS |
| 39 | Male | 63 | 1 | 1 | 0 | 3 | MSS |
| 40 | Female | 24 | 3 | 0 | 0 | 2 | MSI |
| 41 | Female | 28 | 1 | 0 | 0 | 1 | MSI |
| 42 | Female | 34 | 2 | 0 | 0 | 1 | MSI |
| 43 | Female | 34 | 4 | 1 | 0 | 3 | MSI |
| 44 | Male | 35 | 3 | 0 | 0 | 2 | MSI |
| 45 | Male | 37 | 2 | 0 | 0 | 1 | MSI |
| 46 | Male | 37 | 3 | 0 | 0 | 2 | MSI |
| 47 | Male | 40 | 3 | 0 | 0 | 2 | MSI |
| 48 | Male | 42 | 3 | 0 | 0 | 2 | MSI |
| 49 | Male | 42 | 4 | 2 | 1 | 4 | MSI |
| 50 | Female | 47 | 1 | 0 | 0 | 1 | MSI |
| 51 | Male | 48 | 3 | 0 | 0 | 2 | MSI |
| 52 | Male | 48 | 3 | 1 | 0 | 3 | MSI |
| 53 | Female | 49 | 3 | 1 | 0 | 3 | MSI |
| 54 | Female | 50 | 2 | 0 | 1 | 4 | MSI |
| 55 | Male | 54 | 3 | 0 | 0 | 2 | MSI |
| 56 | Male | 59 | 4 | 0 | 0 | 2 | MSI |
| 57 | Male | 65 | 3 | 1 | 0 | 3 | MSI |
| 58 | Female | 71 | 3 | 1 | 0 | 3 | MSI |
| 59 | Male | 72 | 3 | 0 | 0 | 2 | MSI |
| 60 | Female | 29 | 4 | 0 | 0 | 2 | MSS |
| 61 | Male | 32 | 4 | 0 | 0 | 2 | MSS |
| 62 | Female | 32 | 3 | 2 | 1 | 4 | MSS |
| 63 | Female | 35 | 3 | 2 | 1 | 4 | MSS |
| 64 | Female | 38 | 2 | 0 | 0 | 1 | MSS |
| 65 | Female | 38 | 3 | 0 | 0 | 2 | MSS |
| 66 | Male | 39 | 2 | 0 | 0 | 1 | MSS |
| 67 | Female | 41 | 3 | 0 | 0 | 2 | MSS |
| 68 | Male | 42 | 4 | 0 | 0 | 2 | MSS |
| 69 | Male | 42 | 3 | 0 | 0 | 2 | MSS |
| 70 | Female | 44 | 4 | 2 | 1 | 4 | MSS |
| 71 | Male | 45 | 3 | 0 | 0 | 2 | MSS |
| 72 | Male | 48 | 3 | 0 | 0 | 2 | MSS |
| 73 | Female | 52 | 3 | 2 | 1 | 4 | MSS |
| 74 | Male | 54 | 3 | 0 | 0 | 2 | MSS |
| 75 | Male | 55 | 3 | 0 | 0 | 2 | MSS |
| 76 | Female | 58 | 2 | 0 | 0 | 1 | MSS |
| 77 | Male | 58 | 3 | 0 | 0 | 2 | MSS |
| 78 | Male | 62 | 3 | 0 | 0 | 2 | MSS |
| 79 | Male | 62 | 4 | 0 | 0 | 2 | MSS |
| 80 | Female | 64 | 3 | 1 | 0 | 3 | MSS |

**Supplementary Table 2**. Patients’ clinicopathological features in 72 colorectal cancer informative cases.

| ID | Sex | Age | T  stage | N  stage | M  stage | TNM stage | MSI status | Recurrence | Status | DFS time | OS time | ELMO1 Score |
| --- | --- | --- | --- | --- | --- | --- | --- | --- | --- | --- | --- | --- |
| 1 | Male | 48 | T3 | N0 | M0 | II | MSS | YES | ALIVE | 22.6 | 22.6 | 2 |
| 2 | Male | 33 | T3 | N0 | M0 | II | MSI | NO | ALIVE | 50.8 | 50.8 | 9 |
| 3 | Female | 64 | T3 | N1 | M0 | III | MSS | YES | DEAD | 15.8 | 15.8 | 1 |
| 4 | Female | 71 | T3 | N1 | M0 | III | MSI | NO | DEAD | 52.2 | 52.2 | 4 |
| 5 | Male | 72 | T3 | N0 | M0 | II | MSS | NO | DEAD | 52.5 | 52.5 | 1 |
| 6 | Male | 54 | T3 | N0 | M0 | II | MSI | NO | ALIVE | 52.8 | 52.8 | 2 |
| 7 | Female | 38 | T2 | N0 | M0 | I | MSS | NO | ALIVE | 33.9 | 33.9 | 1 |
| 8 | Male | 53 | T3 | N0 | M0 | II | MSI | NO | ALIVE | 40.3 | 40.3 | 12 |
| 9 | Female | 53 | T4 | N1 | M1 | IV | MSI | YES | DEAD | 4.5 | 4.5 | 12 |
| 10 | Female | 41 | T3 | N0 | M0 | II | MSS | NO | ALIVE | 55.8 | 55.8 | 2 |
| 11 | Female | 30 | T2 | N0 | M0 | I | MSS | YES | DEAD | 24.9 | 31.7 | 2 |
| 12 | Male | 63 | T3 | N0 | M0 | II | MSI | NO | ALIVE | 44.2 | 44.2 | 8 |
| 13 | Male | 40 | T4 | N0 | M0 | II | MSI | NO | ALIVE | 43.0 | 43.0 | 2 |
| 14 | Female | 49 | T3 | N1 | M0 | III | MSI | NO | DEAD | 48.2 | 48.2 | 9 |
| 15 | Female | 48 | T1 | N0 | M0 | I | MSS | NO | ALIVE | 41.2 | 41.2 | 6 |
| 16 | Female | 38 | T2 | N0 | M0 | I | MSI | NO | ALIVE | 24.6 | 24.6 | 8 |
| 17 | Male | 54 | T3 | N0 | M0 | II | MSI | NO | ALIVE | 47.7 | 47.7 | 6 |
| 18 | Female | 34 | T4 | N1 | M0 | III | MSI | YES | DEAD | 30.5 | 50.6 | 2 |
| 19 | Female | 47 | T1 | N0 | M0 | I | MSI | YES | DEAD | 39.0 | 39.0 | 6 |
| 20 | Female | 35 | T3 | N2 | M1 | IV | MSS | YES | DEAD | 17.5 | 17.5 | 1 |
| 21 | Female | 34 | T2 | N0 | M0 | I | MSI | NO | ALIVE | 31.1 | 31.1 | 6 |
| 22 | Male | 54 | T3 | N0 | M0 | II | MSS | NO | ALIVE | 48.5 | 48.5 | 6 |
| 23 | Female | 33 | T3 | N0 | M1 | IV | MSI | NO | DEAD | 21.2 | 21.2 | 9 |
| 24 | Female | 40 | T3 | N0 | M0 | II | MSS | NO | DEAD | 42.7 | 42.7 | 2 |
| 25 | Male | 39 | T3 | N1 | M0 | III | MSS | NO | DEAD | 53.8 | 53.8 | 6 |
| 26 | Male | 65 | T3 | N1 | M0 | III | MSI | NO | DEAD | 55.0 | 55.0 | 8 |
| 27 | Female | 24 | T3 | N0 | M0 | II | MSI | YES | DEAD | 35.5 | 35.5 | 9 |
| 28 | Male | 35 | T3 | N0 | M0 | II | MSI | YES | DEAD | 52.8 | 52.8 | 12 |
| 29 | Male | 62 | T4 | N0 | M0 | II | MSS | YES | DEAD | 16.3 | 30.2 | 1 |
| 30 | Male | 63 | T3 | N0 | M0 | II | MSI | NO | ALIVE | 48.7 | 48.7 | 6 |
| 31 | Female | 41 | T3 | N0 | M0 | II | MSI | NO | ALIVE | 48.9 | 48.9 | 9 |
| 32 | Female | 28 | T1 | N0 | M0 | I | MSI | NO | ALIVE | 25.0 | 25.0 | 3 |
| 33 | Male | 55 | T3 | N0 | M0 | II | MSS | NO | ALIVE | 47.6 | 47.6 | 3 |
| 34 | Female | 42 | T3 | N0 | M0 | II | MSI | NO | ALIVE | 36.4 | 36.4 | 9 |
| 35 | Male | 38 | T3 | N0 | M0 | II | MSS | NO | ALIVE | 44.8 | 44.8 | 1 |
| 36 | Male | 40 | T4 | N0 | M0 | II | MSI | NO | ALIVE | 52.5 | 52.5 | 12 |
| 37 | Male | 72 | T3 | N0 | M0 | II | MSI | NO | ALIVE | 50.9 | 50.9 | 8 |
| 38 | Female | 52 | T3 | N2 | M1 | IV | MSS | NO | DEAD | 30.5 | 30.5 | 4 |
| 39 | Male | 35 | T3 | N0 | M0 | II | MSS | NO | ALIVE | 48.9 | 48.9 | 1 |
| 40 | Male | 59 | T4 | N0 | M0 | II | MSI | NO | ALIVE | 52.6 | 52.6 | 6 |
| 41 | Female | 29 | T4 | N0 | M0 | II | MSS | NO | ALIVE | 36.1 | 36.1 | 1 |
| 42 | Male | 62 | T3 | N0 | M0 | II | MSS | NO | ALIVE | 47.5 | 47.5 | 1 |
| 43 | Female | 53 | T3 | N1 | M1 | IV | MSS | YES | DEAD | 40.3 | 61.7 | 2 |
| 44 | Male | 28 | T4 | N2 | M0 | III | MSS | YES | DEAD | 36.2 | 36.2 | 12 |
| 45 | Male | 42 | T3 | N0 | M0 | II | MSS | NO | ALIVE | 56.3 | 56.3 | 1 |
| 46 | Male | 48 | T3 | N0 | M0 | II | MSI | NO | ALIVE | 54.9 | 54.9 | 2 |
| 47 | Male | 37 | T3 | N0 | M0 | II | MSI | NO | ALIVE | 46.7 | 46.7 | 2 |
| 48 | Male | 54 | T3 | N0 | M0 | II | MSS | YES | DEAD | 45.3 | 45.3 | 4 |
| 49 | Male | 39 | T2 | N0 | M0 | I | MSS | NO | ALIVE | 24.5 | 24.5 | 4 |
| 50 | Male | 42 | T4 | N2 | M1 | IV | MSI | YES | DEAD | 4.4 | 4.4 | 9 |
| 51 | Female | 39 | T3 | N0 | M0 | II | MSI | YES | ALIVE | 50.3 | 50.3 | 9 |
| 52 | Male | 48 | T3 | N1 | M0 | III | MSI | NO | DEAD | 49.1 | 49.1 | 9 |
| 53 | Male | 42 | T3 | N0 | M0 | II | MSS | YES | ALIVE | 31.5 | 31.5 | 1 |
| 54 | Male | 42 | T4 | N0 | M0 | II | MSS | NO | ALIVE | 51.2 | 51.2 | 1 |
| 55 | Male | 45 | T3 | N0 | M0 | II | MSS | NO | ALIVE | 52.9 | 52.9 | 2 |
| 56 | Male | 41 | T3 | N0 | M0 | II | MSS | NO | ALIVE | 47.9 | 47.9 | 4 |
| 57 | Female | 59 | T2 | N0 | M0 | I | MSI | NO | ALIVE | 23.8 | 23.8 | 8 |
| 58 | Female | 52 | T3 | N0 | M1 | IV | MSS | NO | DEAD | 27.5 | 27.5 | 9 |
| 59 | Male | 40 | T3 | N0 | M0 | II | MSI | NO | ALIVE | 51.9 | 51.9 | 9 |
| 60 | Male | 32 | T4 | N0 | M0 | II | MSS | NO | ALIVE | 52.8 | 52.8 | 1 |
| 61 | Male | 58 | T3 | N0 | M0 | II | MSS | NO | ALIVE | 55.4 | 55.4 | 2 |
| 62 | Male | 40 | T3 | N1 | M1 | IV | MSI | YES | DEAD | 20.2 | 20.2 | 2 |
| 63 | Male | 37 | T2 | N0 | M0 | I | MSI | NO | ALIVE | 39.5 | 39.5 | 8 |
| 64 | Female | 46 | T3 | N1 | M0 | III | MSS | NO | ALIVE | 62.8 | 62.8 | 4 |
| 65 | Female | 32 | T3 | N2 | M1 | IV | MSS | NO | DEAD | 6.9 | 6.9 | 2 |
| 66 | Male | 42 | T3 | N0 | M0 | II | MSI | NO | ALIVE | 48.4 | 48.4 | 6 |
| 67 | Female | 38 | T3 | N0 | M0 | II | MSS | NO | ALIVE | 51.0 | 51.0 | 2 |
| 68 | Female | 50 | T2 | N0 | M1 | IV | MSI | YES | DEAD | 38.5 | 43.7 | 12 |
| 69 | Male | 71 | T3 | N1 | M0 | III | MSS | YES | DEAD | 38.8 | 48.8 | 1 |
| 70 | Female | 34 | T2 | N0 | M0 | I | MSS | YES | DEAD | 39.1 | 39.1 | 1 |
| 71 | Female | 58 | T2 | N0 | M0 | I | MSS | NO | ALIVE | 47.7 | 47.7 | 6 |
| 72 | Female | 44 | T4 | N2 | M1 | IV | MSS | YES | DEAD | 2.9 | 60.9 | 8 |
